# Spatiotemporal epidemiology of substance-related accidental acute toxicity deaths in Canada from 2016 to 2017

**DOI:** 10.1186/s12889-024-18883-2

**Published:** 2024-06-20

**Authors:** Mohammad Howard-Azzeh, Rania Wasfi, Tanya Kakkar, Mallory Flynn, Jenny Rotondo, Emily Schleihauf, Matthew Bowes, Erin E. Rees

**Affiliations:** 1 Health Promotion and Chronic Disease Prevention Branch, Substance-Related Harms Division, Public Health Agency of Canada, Ontario, Canada; 2 Department of Population Medicine, Ontario Veterinary College, University of Guelph, Ontario, Canada; 3 Public Health Risk Sciences Division, National Microbiology Laboratory, Public Health Agency of Canada, Quebec, Canada; 4 Department of Statistics, University of British Columbia, British Columbia, Canada; 5 Canadian Public Health Service, Public Health Agency of Canada, Ontario, Canada; 6 Nova Scotia Medical Examiner Service, Department of Justice, Nova Scotia, Canada; 7 Department of Pathology and Microbiology, Faculty of Veterinary Medicine, University of Montreal, Quebec, Canada

**Keywords:** Acute toxicity, Canada, Chart review study, Coroner, Death investigations, Drug, Overdose, Medical examiner, Mortality, Poisoning, SaTScan, Spatial scan statistic, Epidemiology, Spatiotemporal, Geographic distribution

## Abstract

**Objectives:**

In Canada, substance-related accidental acute toxicity deaths (AATDs) continue to rise at the national and sub-national levels. However, it is unknown if, where, when, and to what degree AATDs cluster in space, time, and space–time across the country. The objectives of this study were to 1) assess for clusters of AATDs that occurred in Canada during 2016 and 2017 at the national and provincial/territorial (P/T) levels, and 2) examine the substance types detected in AATD cases within each cluster.

**Methods:**

Two years of person-level data on AATDs were abstracted from coroner and medical examiner files using a standardized data collection tool, including the decedent's postal code and municipality information on the places of residence, acute toxicity (AT) event, and death, and the substances detected in the death. Data were combined with Canadian census information to create choropleth maps depicting AATD rates by census division. Spatial scan statistics were used to build Poisson models to identify clusters of high rates (*p* < 0.05) of AATDs at the national and P/T levels in space, time, and space–time over the study period. AATD cases within clusters were further examined for substance types most present in each cluster.

**Results:**

Eight clusters in five regions of Canada at the national level and 24 clusters in 15 regions at the P/T level were identified, highlighting where AATDs occurred at far higher rates than the rest of the country. The risk ratios of identified clusters ranged from 1.28 to 9.62. Substances detected in clusters varied by region and time, however, opioids, stimulants, and alcohol were typically the most commonly detected substances within clusters.

**Conclusion:**

Our findings are the first in Canada to reveal the geographic disparities in AATDs at national and P/T levels using spatial scan statistics. Rates associated with substance types within each cluster highlight which substance types were most detected in the identified regions. Findings may be used to guide intervention/program planning and provide a picture of the 2016 and 2017 context that can be used for comparisons of the geographic distribution of AATDs and substances with different time periods.

**Supplementary Information:**

The online version contains supplementary material available at 10.1186/s12889-024-18883-2.

## Introduction

In the last few decades, there have been substantial shifts in drug use patterns and the regulation of drugs in Canada [1, 2]. These shifts are both the cause and response to the continual increase in overall drug use, the types of drugs used and circulating in the illegal drug supply, and the harms related to drug use [2, 3]. Increases in drug use and related harms in Canada have been influenced by a range of factors, including changes in prescription patterns, legislation, drug availability and increasing toxicity of the drug supply, and social attitudes towards drug use [2–6]. Substance types like opioids, stimulants, alcohol, and benzodiazepines have been commonly detected in acute toxicity deaths (ATDs, sometimes called ‘overdose’ or ‘poisoning’ deaths) across Canada [7, 8]. In 2017, ATDs were responsible for approximately 1.6% of all deaths in Canada and continue to rise [7–10]. Accidental opioid-related ATDs alone roughly tripled from 2016 to 2022, with the largest increase occurring in 2020 [8]. The World Health Organization has shown that in 2019 Canada may have had the second-highest rate of ATDs in the world [11]. Yet, much is to be understood regarding the distribution of ATDs across Canada.

Rates of ATD vary substantially between provinces/territories (P/Ts), indicating that ATDs in Canada are not evenly distributed across the country [12, 13]. Spatiotemporal analyses of data from British Columbia (BC) and Alberta (AB) have demonstrated spatial clustering of different drug-related outcomes within their respective provinces [14–18]. For example, Hu et al. found that the probability of surviving an acute toxicity event was lower in rural areas compared to urban areas of BC, possibly due to a lack of access to harm reduction services in rural communities [14]. They also found a statistically significant region of higher risk in southern BC that included the areas around Kamloops, Kelowna, Merit, and many of the communities along the Fraser River [14]. They further identified a region of lower risk in Greater Vancouver, potentially due to the positive effects of local intervention methods [14, 19, 20]. Despite findings that suggest that ATDs cluster in space, to date, there are no spatiotemporal analyses on ATDs at the national level or comprehensive comparisons across multiple P/Ts when analysed at the P/T level in Canada.

Spatial scan statistics are amongst the most widely used and understood methods for the detection of geographic clusters in disease surveillance [21]. Using these methods to examine the spatial distribution of different substance-related outcomes in different populations in the entire United States, studies have identified and quantified clusters in regions where drug-related outcomes occurred at higher than expected rates/proportions [22–24]. These studies were largely in agreement with each other and offer a comprehensive understanding of clustering and the distribution of disease across entire populations. These same techniques may provide valuable information to support the development of targeted interventions in Canada.

Data regarding the location, time, and substances involved among substance-related ATDs that took place in Canada between 2016 and 2017 were collected as part of a retrospective national chart review study led by the Public Health Agency of Canada (Rotondo J, VanSteelandt A, Kouyoumdjian F, Bowes MJ, Kakkar T, Jones G, et al.: Substance-related acute toxicity deaths in Canada from 2016 to 2017: A protocol for a retrospective chart review study of coroner and medical examiner files, forthcoming). With this information, it was possible to begin identifying geographic trends and risk factors associated with substance-related ATD at the national level. Therefore, the objectives of this study were to i) analyse this data using spatial scan statistics to identify clusters of high mortality rates in space, time, and space–time for all substance-related accidental acute toxicity deaths (AATD) at the national and at P/T levels and ii) further characterize identified clusters by examining the substance type-specific AATD rates based on substances detected in toxicology. Results from this study will establish the foundational spatiotemporal epidemiology of AATDs in Canada, which can help guide the development of interventions by identifying regions most affected by AATDs and the type of substances involved, to establish a baseline in AATD trends from which we can both assess the evolution of the overdose crisis over time, and evaluate the impact of intervention strategies moving forward.

## Methods

### Data

Data for this study were obtained from a retrospective chart review of the coroner and medical examiner files of people who died from substance-related acute toxicity (AT) between 2016 and 2017 in Canada (Rotondo J, VanSteelandt A, Kouyoumdjian F, Bowes MJ, Kakkar T, Jones G, et al.: Substance-related acute toxicity deaths in Canada from 2016 to 2017: A protocol for a retrospective chart review study of coroner and medical examiner files, forthcoming). The people included in this study were any individuals who died of a substance-related accidental AT event between January 1st, 2016 and December 31st, 2017. Data collection in BC differed slightly, where people in BC were included in the study if they died of a substance-related accidental AT event involving unregulated or pharmaceutical substances not prescribed to them (i.e. information on AATDs only involving prescribed substances or alcohol was not available). Substance type involvement for the purposes of this study was based on the detection of substances in post-mortem toxicological screening and may not necessarily be limited to substances that directly caused death, with the exception of BC, where only the substances that were thought to have contributed to death were reported. A standardized data collection tool was used to abstract case information, including information on the manner of death, the substances involved, and the postal code or municipality of residence, the AT event, and death. The chart review study's protocol has been described in detail elsewhere (Rotondo J, VanSteelandt A, Kouyoumdjian F, Bowes MJ, Kakkar T, Jones G, et al.: Substance-related acute toxicity deaths in Canada from 2016 to 2017: A protocol for a retrospective chart review study of coroner and medical examiner files, forthcoming). This spatiotemporal study included only ATDs for which the manner of death was accidental and did not include data from ATDs for which the manner of death was suicide or undetermined.

Spatiotemporal analyses were conducted using the census division (CD) and the centroid of the census subdivision (CSD) of the residence of the person who died. This was determined based on the residential postal code. If the residential postal code was not available, the location was assigned using the centroid of the residential municipality. If the residential municipality was not available, the postal code or municipality of the AT event location was used (*n* = 126). When residence and AT event locations were not available, the postal code or municipality of the death location was used (*n* = 141). Statistics Canada’s Postal Code Conversion File Plus (PCCF +) residential file version 7B was used to identify the CSD of each postal code [25]. Mortality rates were calculated using Statistics Canada's July 2016 and 2017 CD and CSD population estimates as denominators. To protect privacy, all counts using chart review study data were randomly rounded to base three and all counts less than 10 have been suppressed. Proportions and rates are based on rounded counts. All of the data was used in the analyses, information was only suppressed in the presentation of data and did not affect the analysis in any ways.

Although residence, event, and death locations were largely in agreement with each other at the CSD level, there were some discrepancies between these variables. 73% of the residence and AT event locations had the same CSD reported, while 9% had different CSDs reported and 18% had either the residence or the AT event locations missing. 81% of the residence and death locations had the same CSD reported, while 13% had different CSDs reported and 7% either the home location or the death location was missing. Lastly, 79% of the AT event and acute death locations had the same CSD reported, while 5% had different CSDs reported and 15% had either the death location or the AT event location missing.

### Descriptive analyses

AATD mortality rates were calculated by CSD, CD, P/T, and detected substance types. Only the six most commonly detected substance type categories were examined in this analysis, including opioids, stimulants, alcohol, benzodiazepines, antidepressants, and antipsychotics. A breakdown of the substances included in each category is available in Supplementary Table 1. Analyses were conducted using Stata 17 (StataCorp, College Station, TX).

To provide context for interpreting results from the spatial scan statistic choropleth maps depicting the AATD mortality rates by CD were created using ArcGIS v10 [26]. Although CSDs were used to identify clusters, choropleth maps depict AATD rates across CDs rather than CSDs as this resulted in fewer regions with suppressed rates and greater visual clarity.

### Spatiotemporal analyses

Scan statistics using Poisson models were used to identify AATD clusters in space, time, and space–time [21]. As input, case locations were defined by the CSD centroid, and AATD mortality rates were calculated using the CSD population as the denominator. A national-level cluster was defined as a cluster identified using data from the entire country and compared to the national mean mortality rate, while a P/T-level cluster was defined as a cluster identified using information from a given P/T and compared to that respective P/T's mean mortality rate. To identify clusters at the national and P/T levels, scan statistics were performed for all of Canada and then on each P/T individually. Due to relatively few AATDs, data for Yukon, Northwest Territories, and Nunavut, herein called the Territories, were combined for the P/T-level analyses.

A maximum cluster spatial scanning window of 50% of the population at risk was used since there were no a priori assumptions regarding the maximum cluster size. Furthermore, no maximum value was set for the radius of the scanning window since deaths were not evenly distributed across Canada. These parameters allow for the identification of large and small clusters and minimize pre-selection bias [21]. Each scan was performed using the entire study period (i.e., January 1st, 2016 to December 31st, 2017) using a maximum temporal window of 50% (i.e., 1 year). Standard Monte Carlo estimations with 999 replications were used for all scans. Days were used for the smallest temporal resolution for purely temporal scans; however, due to computational limitations, months were used for the smallest temporal resolution of space–time scans. Spatial clusters were not allowed to overlap in space, temporal clusters were not allowed to overlap in time. Space–time clusters were allowed to overlap in space or time, but not both space and time. Crude rates were used in this study to identify where AATDs occurred at the highest rates, without adjustment for explanatory variables.

All scan statistics were performed using SaTScan v 9.6 with a one-tailed hypothesis (α = 0.05), identifying clusters of CSDs with higher-than-expected mortality rates [21]. Each cluster's p-values, observed to expected ratios, radius, location in space and time, and member CSDs were reported. Statistically significant space and space–time clusters were mapped using ArcGIS v10.

## Results

**Fig. 1:**
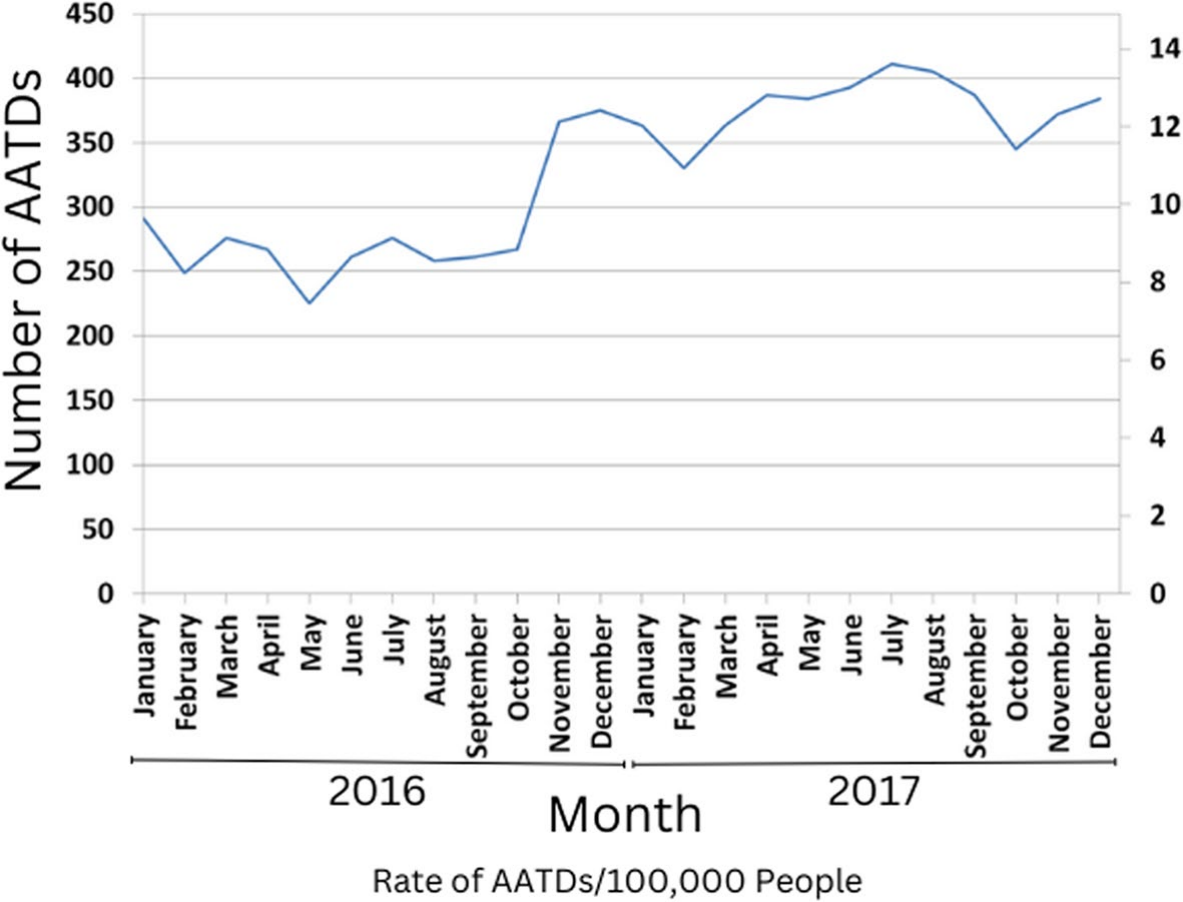
Frequency^a^ of accidental acute toxicity deaths (AATDs) by month in Canada, January 1st, 2016 to December 31st, 2017 Notes: ^a^ Counts were based on counts randomly rounded to base 3

**Fig. 2:**
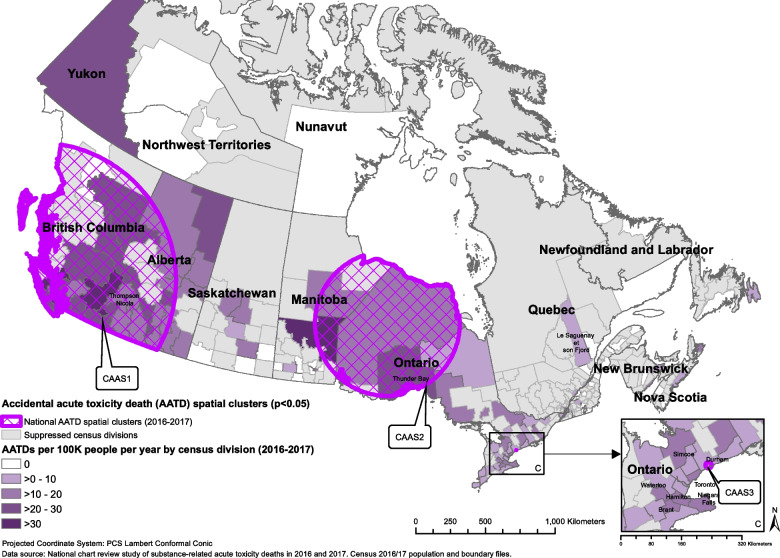
Locations of statistically significant national-level spatial clusters of accidental acute toxicity deaths (AATDs) identified with the spatial scan statistic using Poisson models depicted over a choropleth map illustrating census division AATD rates across Canada, 2016 to 2017

**Fig. 3:**
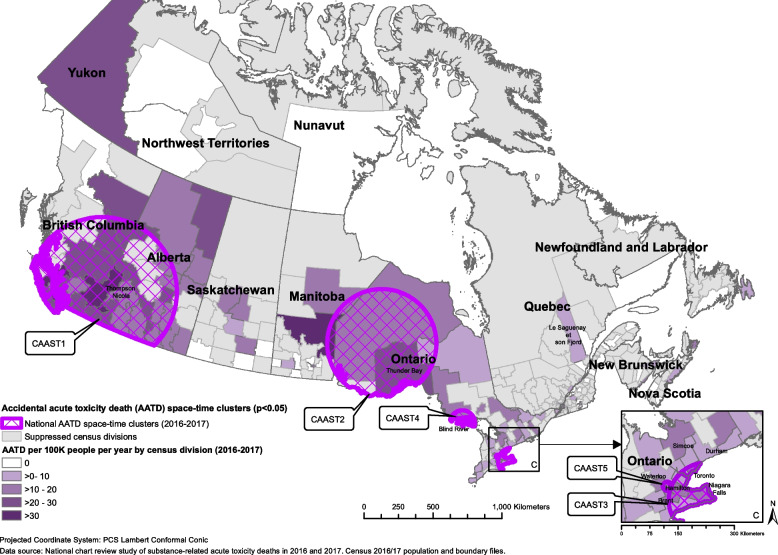
Locations of statistically significant national-level space-time clusters of accidental acute toxicity deaths (AATDs) identified with the spatial scan statistic using Poisson models depicted over a choropleth map illustrating census division AATD rates across Canada, 2016 to 2017

**Fig. 4:**
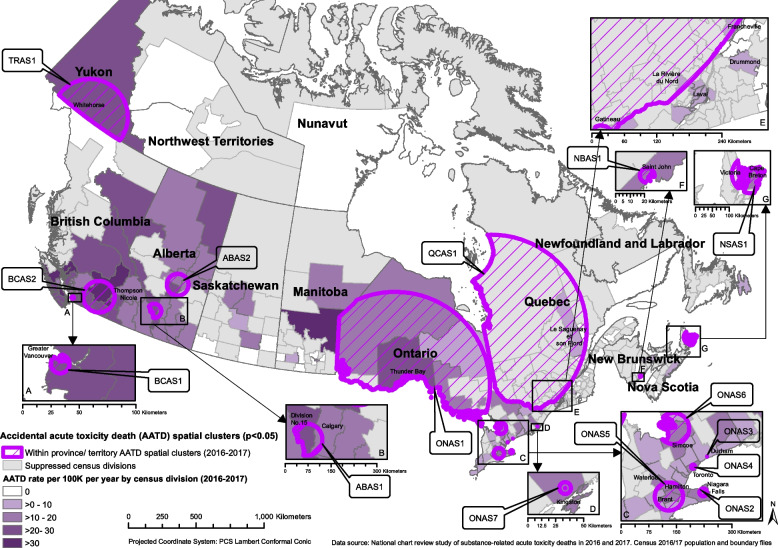
Locations of statistically significant provincial/territorial-level spatial clusters of accidental acute toxicity deaths (AATDs) identified with the spatial scan statistic using Poisson models depicted over a choropleth map illustrating census division AATD rates across Canada, 2016 to 2017

**Fig. 5:**
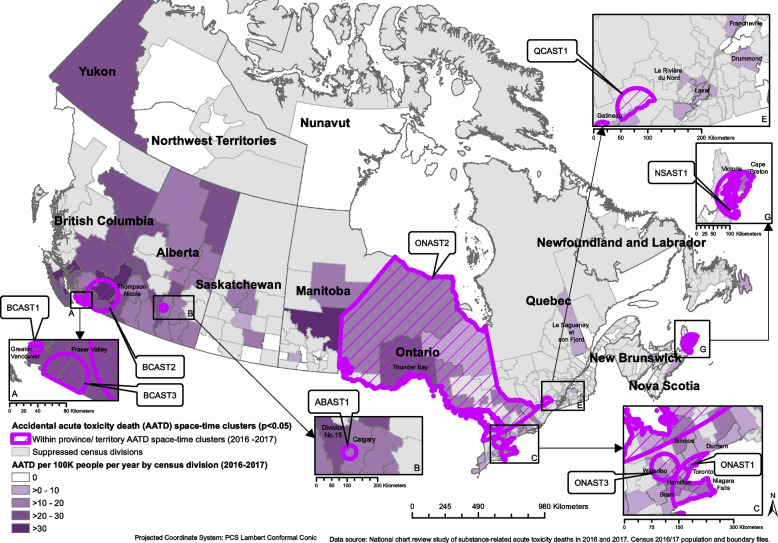
Locations of statistically significant provincial/territorial-level space–time clusters of accidental acute toxicity deaths (AATDs) identified with the spatial scan statistic using Poisson models depicted over a choropleth map illustrating census division AATD rates across Canada, 2016 to 2017

Of the 7,902 AATDs that occurred in Canada in 2016 and 2017, 7,899 had location information and were included in this analysis. The average mortality rate from AATDs across Canada during this period was 10.9 AATDs per 100,000 people per year (Table 1). AATDs generally increased throughout the study period, with the highest number of deaths occurring in July 2017 (Fig. 1). Opioids were detected most often in these deaths, followed by stimulants, alcohol, then benzodiazepines (Table 1). More than one substance could be detected in a single death. The order of most commonly detected substance types involved in AATDs varied by province (Table 1). BC, AB, and the Territories had the highest mortality rates at 25.0, 17.9, and 12.7 AATDs per 100,000 people per year, respectively (Table 1). Prince Edward Island (PE), Quebec (QC), and Newfoundland and Labrador (NL) had the lowest mortality rates at 4.0, 4.1, and 4.8 AATDs per 100,000 people per year, respectively (Table 1). As seen in Figs. 2, 3, 4 and 5 and Supplementary Table 2, the CDs with the highest AATD rates occurred in BC, AB, eastern Manitoba (MB), and northwestern Ontario (ON). 36 CDs had no AATDs during this time. The highest rates of AATDs by CD were found in Thompson-Nicola BC, Division No. 19 MB, Strathcona BC, Central Okanagan BC, and Nanaimo BC, at more than 30 AATDs per 100,000 people per year (S. Table 2).

**Table 1:** Number^a^ and rates^a^ of accidental acute toxicity deaths (AATDs) in Canada by substance types detected and region, 2016 to 2017

Region	Average population^b^	Total (rate)	Opioid (rate)	Fentanyl opioid (rate)	Non-fentanyl opioid (rate)	Stimulant (rate)	Alcohol (rate)	Benzodiazepine (rate)	Antidepressant (rate)	Antipsychotic (rate)	Polysubstance (rate)
Canada	36,330,221	7,899 (10.9)	6,279 (8.6)	4,152 (5.7)	3,762 (5.2)	4,791 (6.6)	2,667 (3.7)	1,989 (2.7)	1,725 (2.4)	759 (1)	5,749 (7.9)
Territories	117,972	30 (12.7)	18 (7.6)	12 (5.1)	12 (5.1)	18 (7.6)	15 (6.4)	sup	sup	sup	14 (5.9)
British Columbia	4,894,413	2,445 (25)	2,040 (20.8)	1,839 (18.8)	804 (8.2)	1,665 (17)	615 (6.3)	105 (1.1)	111 (1.1)	54 (0.6)	2441 (24.9)
Alberta	4,218,642	1509 (17.9)	1,281 (15.2)	918 (10.9)	714 (8.5)	882 (10.5)	537 (6.4)	468 (5.5)	411 (4.9)	171 (2.0)	920 (10.9)
Saskatchewan	1,143,179	204 (8.9)	153 (6.7)	24 (1)	138 (6)	99 (4.3)	72 (3.1)	60 (2.6)	60 (2.6)	30 (1.3)	104 (4.5)
Manitoba	1,324,493	261 (9.9)	180 (6.8)	78 (2.9)	135 (5.1)	123 (4.6)	126 (4.8)	111 (4.2)	93 (3.5)	30 (1.1)	114 (4.3)
Ontario	13,973,029	2,490 (8.9)	1,995 (7.1)	1,134 (4.1)	1,407 (5)	1,437 (5.1)	996 (3.6)	753 (2.7)	651 (2.3)	264 (0.9)	1578 (5.6)
Quebec	8,269,540	672 (4.1)	396 (2.4)	108 (0.7)	351 (2.1)	369 (2.2)	225 (1.4)	318 (1.9)	288 (1.7)	174 (1.1)	403 (2.4)
New Brunswick	764,990	90 (5.9)	69 (4.5)	12 (0.8)	63 (4.1)	51 (3.3)	21 (1.4)	54 (3.5)	51 (3.3)	18 (1.2)	57 (3.7)
Nova Scotia	946,459	135 (7.1)	102 (5.4)	15 (0.8)	93 (4.9)	99 (5.2)	42 (2.2)	84 (4.4)	39 (2.1)	12 (0.6)	89 (4.7)
Prince Edward Island	148,690	12 (4)	sup	0 (0)	sup	sup	sup	sup	sup	0 (0)	sup
Newfoundland	528,836	51 (4.8)	39 (3.7)	12 (1.1)	33 (3.1)	36 (3.4)	15 (1.4)	21 (2)	15 (1.4)	0 (0)	23 (2.2)

^a^Counts and mortality rates are based on counts randomly rounded to base 3, and numbers less than 10 have been suppressed (sup). Mortality rates in brackets are equal to the number of AATDs per 100,000 population per year

^b^Population size is the average from 2016 and 2017. More than one substance type could be detected from a single AATD

**Table 2:** Characteristics^a^ of national and provincial/territorial-level temporal clusters^b^ of accidental acute toxicity deaths (AATDs) in Canada, 2016 to 2017

Cluster name	Region	Time frame	Risk ratio	Observed AATDs	Expected AATDs	*P*-value	O/E^c^	Observed rate	Expected rate
CAAT	Canada	2016/12/13 to 2017/12/4	1.34	4,452	3,873	< 0.001	1.15	12.5	10.9
BCAT	British Columbia	2016/11/10 to 2017/8/26	1.80	1,329	973	< 0.001	1.37	34.1	25.0
ABAT	Alberta	2017/11/30 to 2017/12/1	4.62	18	4	0.002	4.50	81.8	17.9
MBAT	Manitoba	2016/9/29 to 2017/9/13	1.70	159	126	0.044	1.26	12.6	9.9
ONAT	Ontario	2017/4/28 to 2017/12/31	1.50	1,089	850	< 0.001	1.28	11.4	8.9

^a^Counts and mortality rates are based on counts randomly rounded to base 3, and numbers less than 10 have been suppressed (sup). Mortality rates are equal to the number of AATDs per 100,000 population per year

^b^Purely temporal AATD clusters were identified with the spatial scan statistic using Poisson models at the national and provincial/territorial levels

^c^O/E = Observed over expected AATDs

### Temporal clusters

The national-level temporal cluster analysis detected one large temporal cluster in the second half of the study period from December 13th, 2016 to December 4th, 2017 (CAAT) (Table 2). P/T-level temporal clustering was observed from November 10th, 2016 to July 26th, 2017 in BC (BCAT), November 30th, 2017 to December 1st, 2017 in AB (ABAT), September 29th, 2016 to September 13th, 2017 in MB (MBAT), and April 28th, 2017 to December 31st, 2017 in ON (ONAT). Temporal clusters had risk ratios ranging from 1.34 (CAAT) to 4.62 (ABAT). It should be noted that the period of cluster ABAT was 1 day, leading to an over-estimated risk ratio.

In each temporal cluster, when comparing AATD rates by substance type with their respective regions, there were generally higher rates of total AATDs, opioids, fentanyl-opioids, non-fentanyl opioids, stimulants, alcohol, but generally the same rates of benzodiazepines, antidepressants, and antipsychotics (Table 3). However, cluster ABAT had higher rates of all substance types. AATD rates of detected fentanyl opioids and stimulants were particularly high in the BCAT and ABAT clusters. The high values of cluster ABAT were in part due to the short period.

**Table 3:** Detected substance type-specific accidental acute toxicity death (AATD) rates^a^ among national and provincial/territorial-level temporal AATD clusters in Canada, 2016 to 2017

Cluster	All AATDs	Opioids	Fentanyl opioids	Non-fentanyl opioids	Stimulants	Alcohol	Benzodiazepines	Antidepressants	Antipsychotics
CAAT	22.6	10.3	7.5	5.5	8.0	4.1	2.9	2.4	1.2
BCAT	34.3	29.5	28.1	9.3	23.1	8.5	1.3	1.5	0.8
ABAT	155.7	155.7	155.7	sup	103.8	sup	sup	sup	sup
MBAT	12.6	8.8	4.5	5.9	6.4	6.6	5.7	4.5	1.2
ONAT	11.5	9.7	6.7	6.2	7.0	4.7	2.9	2.1	1.1

^a^Mortality rates are based on counts are randomly rounded to base 3. Mortality rates are equal to the number of AATDs per 100,000 population per year. More than one substance type could be detected from a single AATD

### National-level space and space–time clusters

The spatial scan at the national level identified three statistically significant spatial clusters, where CSD mortality rates were higher than the national average (Fig. 2, Table 4). One large area cluster in western Canada covered most of BC and western AB (CAAS1) while another encompassed eastern MB and northwestern ON, including Thunder Bay, ON (CAAS2). A small area cluster was identified encompassing Oshawa, ON. These clusters had risk ratios ranging from 1.95 (CAAS3) to 3.08 (CAAS1) (Table 4).

**Table 4:** Characteristics^a^ of national and provincial/territorial-level spatial clusters of accidental acute toxicity deaths (AATDs) in Canada, 2016 to 2017

**Cluster**	**Level**	**Relative risk**	**O/E**^**b**^	**Observed**	**Expected**	***P*-value**	**Latitude**	**Longitude**	**Radius (km)**	**CSDs included**^d^	**Population in cluster**
CAAS1	Canada	3.08	2.08	3,795	1,825	< 0.001	49.92	-126.65	989.36	945	8,392,919
CAAS2	Canada	2.01	2.00	108	54	< 0.001	53.02	-89.84	525.02	113	249,197
CAAS3	Canada	1.95	1.92	69	36	0.009	43.90	-78.87	0.00	1	165,734
BCAS1	British Columbia	1.89	1.69	567	336	< 0.001	49.25	-123.11	8.30	4	672,364
BCAS2	British Columbia	1.48	1.41	342	242	< 0.001	50.09	-120.76	116.32	152	484,266
ABAS1	Alberta	4.16	3.60	18	5	< 0.001	50.69	-114.88	52.02	7	12,884
ABAS2	Alberta	1.28	1.18	573	485	0.008	52.87	-113.48	81.06	64	1,356,688
ONAS1	Ontario	1.88	1.80	207	115	< 0.001	48.31	-89.27	694.08	203	643,459
ONAS2	Ontario	1.61	1.53	315	206	< 0.001	43.14	-80.35	37.24	13	1,154,303
ONAS3	Ontario	2.41	2.30	69	30	< 0.001	43.90	-78.87	0.00	1	165,734
ONAS4	Ontario	1.34	1.26	636	506	< 0.001	43.73	-79.39	0.00	1	2,841,090
ONAS5	Ontario	1.96	1.89	108	57	< 0.001	43.08	-79.23	10.14	5	319,575
ONAS6	Ontario	1.75	1.74	99	57	0.003	44.71	-79.78	41.57	16	322,254
ONAS7	Ontario	2.07	2.09	48	23	0.023	44.31	-76.46	0.00	1	128,859
QCAS1	Quebec	1.57	1.41	183	130	0.002	51.26	-78.78	705.51	432	1,600,432
NBAS1	New Brunswick	3.20	2.63	21	8	0.014	45.27	-66.06	0.00	1	69,388
NSAS1	Nova Scotia	3.72	2.80	42	15	< 0.001	46.11	-60.19	55.43	5	105,595
TRAS1	Territories	4.26	2.25	18	8	0.012	60.17	-132.71	286.24	24	31,953

^a^Counts and mortality rates are based on counts randomly rounded to base 3, and numbers less than 10 have been suppressed (sup). Mortality rates are equal to the number of AATDs per 100,000 population per year

^b^O/E = The ratio of observed to expected deaths

^d^CSDs included indicates the number of census subdivisions included in each cluster

There were five statistically significant space–time clusters at the national level (Fig. 3, Table 5). The locations of these clusters were similar to those of the purely spatial clusters. Cluster CAAST1 covered the majority of BC and western AB and occurred from November 1st, 2016 to October 31st, 2017. Cluster CAAST2 covered northwestern ON (including Thunder Bay) and occurred from February 1st, 2017 to December 31st, 2017. Cluster CAAST3 covered southeastern ON, including (Niagara Falls, St. Catharines, Hamilton, Brantford, and Toronto) and occurred from June 1st, 2017 to December 31st, 2017. Cluster CAAST4 covered the majority of Manitoulin Island, ON and the areas just north of it and occurred from January 1st, 2017 to April 30th, 2017. Lastly, CAAST5 covered the cities of Kitchener and Cambridge in southern ON and occurred between January 1st, 2017 and December 31st, 2017. These clusters had risk ratios ranging from 1.53 (CAAST3) to 9.72 (CAAST4) (Table 5). Although the risk ratio for CAAST4 was very high, this cluster had relatively few AATDs (*n* = 12).

**Table 5:** Characteristics^a^ of national and provincial/territorial-level spatiotemporal clusters of accidental acute toxicity deaths (AATDs) in Canada, 2016 to 2017

**Cluster**	**Level**	**Time frame**	**Relative risk**	**O/E**^**b**^	**Observed**	**Expected**	**Latitude**	**Longitude**	**Radius (km)**	**Total CSDs**^c^	***P*-value**	**Population in cluster**
CAAST1	Canada	2016–11-01 to 2017–10-31	3.11	2.51	2,256	900	50.99	-120.24	544.11	853	0.001	8,254,250
CAAST2	Canada	2017–02-01 to 2017–12-31	2.74	2.74	63	23	51.49	-90.22	397.80	98	0.001	228,917
CAAST3	Canada	2017–06-01 to 2017–09-30	1.53	1.52	294	194	42.91	-79.19	96.81	25	0.001	5,308,050
CAAST4	Canada	2017–01-01 to 2017–04-30	9.72	12.00	12	1	45.88	-82.97	89.69	33	0.006	37,529
CAAST5	Canada	2017–01-01 to 2017–12-31	2.00	1.95	84	43	43.40	-80.33	11.67	3	0.022	390,113
ABAST1	Alberta	2017–01-01 to 2017–12-31	1.47	1.36	315	231	50.96	-114.35	22.36	2	0.003	1,288,144
BCAST1	British Columbia	2016–11-01 to 2017–06-30	2.90	2.68	297	111	49.25	-123.11	8.30	4	0.001	672,364
BCAST2	British Columbia	2016–10-01 to 2017–08-31	2.07	1.97	219	111	50.09	-120.76	116.32	152	0.001	484,266
BCAST3	British Columbia	2016–12-01 to 2017–05-31	1.52	1.49	204	137	49.11	-122.35	31.52	33	0.009	1,098,445
ONAST1	Ontario	2017–05-01 to 2017–10-31	1.78	1.66	396	238	42.92	-79.03	104.37	24	0.001	5,270,629
ONAST2	Ontario	2016–12-01 to 2017–11-30	1.80	1.73	234	135	54.99	-85.43	1250.19	348	0.001	1,516,847
ONAST3	Ontario	2017–01-01 to 2017–12-31	1.73	1.69	120	71	43.72	-80.39	35.37	16	0.013	789,737
NSAST1	Nova Scotia	2017–01-01 to 2017–12-31	3.83	3.33	30	9	45.70	-60.59	68.48	14	0.001	126,766
QCAST1	Quebec	2016–03-01 to 2017–02-28	3.01	2.77	36	13	45.60	-75.24	35.28	19	0.007	314,984

^a^Counts and measures of association based on based on counts were randomly rounded to base 3

^b^O/E = The ratio of observed to expected deaths

^c^Total CSDs indicates the number of census subdivisions included in the cluster

In each national-level cluster, when comparing AATD rates by substance type within their respective regions, there were generally higher rates of total AATDs for every substance type considered (Table 6). However, in the CAAS2 and CAAST2 clusters (covering northwestern ON and eastern MB), there were relatively low rates of AATDs with fentanyl detected.

**Table 6:** Detected substance type-specific accidental acute toxicity death (AATD) rates^a^ among national-level spatial and spatiotemporal AATD clusters in Canada, 2016 to 2017

Cluster type	Cluster	All AATDs	Opioids	Fentanyl opioids	Non-fentanyl opioids	Stimulants	Alcohol	Benzodiazepines	Antidepressants	Antipsychotics
**Purely Spatial**	CAAS1	22.6	19	16	8.6	14.7	6.5	3.1	2.8	1.2
	CAAS2	21.7	13.8	2.4	13.2	8.4	10.8	5.4	5.4	3
	CAAS3	20.8	18.1	9.1	13.6	10.9	8.1	10.9	8.1	2.7
**Space–time**	CAAST1	27.4	23.5	21.1	9.3	18.1	7.4	3.4	2.8	1.4
	CAAST2	30.2	18.7	4.3	17.2	11.5	15.8	5.7	5.7	4.3
	CAAST3	16.5	14.3	10.9	9.2	11.9	7.5	4.6	3.1	1.5
	CAAST4	98.1	98.1	sup	98.1	sup	sup	sup	sup	sup
	CAAST5	21.6	18.5	15.4	8.5	18.5	6.2	4.6	4.6	sup

^a^Mortality rates are based on counts randomly rounded to base 3. Mortality rates are equal to the number of AATDs per 100,000 population per year. More than one substance type could be detected from a single AATD

### P/T-level space and space–time clusters

The spatial scan at the P/T level identified 15 statistically significant spatial clusters, where CSD mortality rates were higher than the P/T average (Fig. 4, Table 4). In BC there were two clusters that covered greater Vancouver (BCAS1) and southern BC (including Kamloops and Kelowna, BCAS2). In AB, two clusters were identified; one covering Edmonton to Red Deer (ABAS2) and another the area just west of Calgary (ABAS1). Of the seven clusters identified in ON, one occurred in northern Ontario (ONAS1). The remaining clusters occurred in southern Ontario, including Toronto (ONAS4), Oshawa (ONAS3), Kingston (ONAS7), Barrie and the areas north of Barrie (ONAS6), northeastern Niagara (including St. Catharines, Welland, and Niagara Falls, ONAS5), and central southern Ontario (including Hamilton, Kitchener, Brantford, and Woodstock, ONAS2). The final four clusters were detected in northwestern QC (QCAS1), Saint John, New Brunswick (NB) (NBAS1), Cape Breton, Nova Scotia (NS) and its surrounding areas (NSAS1), and southern Yukon (YT) (including Whitehorse, TRAS1). Risk ratios for purely spatial clusters ranged from 1.34 (ONAS4) to 4.26 (TRAS1) (Table 4).

Nine statistically significant space–time clusters were identified, with locations similar to those of the purely spatial clusters (Fig. 5, Table 5). There were three space–time clusters in BC covering the greater Vancouver area between November 1st, 2016 and June 30th, 2017 (BCAST1), southern BC (including Kamloops and Kelowna) between October 1st, 2016 and August 31st, 2017 (BCAST2), and southwestern BC (including Abbotsford) between December 1st, 2016 and May 31st, 2017 (BCAST3). In AB a cluster was detected in Calgary and its surrounding areas between January 1st, 2017 and December 31st, 2017 (ABAST1). Three ON clusters were detected covering southeastern ON (including Niagara Falls, St. Catharines, Hamilton, Brantford, and Toronto) between May 1st, 2017 and October 31st, 2017 (ONAST1), northern ON between December 1st, 2016 and November 30th, 2017 (ONAST2), and Kitchener, Guelph, and the surrounding areas between January 1st, 2017 and December 31st, 2017 (ONAST3). There was also a cluster in southwestern QC which covered Gatineau between March 1st, 2016, and December 31st, 2017 (QCAST1), and a cluster in NS covering Cape Breton and surrounding areas between March 1st, 2016, and February 28th, 2017 (NSAST1). Risk ratios ranged from 1.47 (ABAST1) to 3.83 (NSAST1) (Table 5).

In each P/T-level cluster, when comparing AATD rates by substance type within their respective regions, there were generally higher rates of total AATDs for every substance type considered (Table 7). However, the rates by substance type varied substantially by region, such as fentanyl opioids commonly detected in western regions but were rare in eastern regions (Table 7). Therefore, the substance types most detected in AATDs at the P/T level were typically similar to the substance types most detected in clusters within their respective P/T, but at much higher rates within the clusters. A list of CSDs included in each cluster of this study is presented in supplementary Table 3.

**Table 7:** Detected substance type-specific accidental acute toxicity death (AATD) rates^a^ among provincial/territorial-level spatial and spatiotemporal AATD clusters in Canada, 2016 to 2017

Cluster type	Cluster	All AATDs	Opioids	Fentanyl opioids	Non-fentanyl opioids	Stimulants	Alcohol	Benzodiazepines	Antidepressants	Antipsychotics
**Purely Spatial**	BCAS1	42.2	35	31.2	14.3	31.5	10.9	1.1	1.6	1.3
	BCAS2	35.3	30.4	28.2	8.7	23.2	9.9	1.9	1.5	sup
	ABAS1	69.9	58.2	sup	58.2	sup	sup	58.2	sup	0
	ABAS2	21	17.6	12.1	9.4	11.5	7.1	7.2	6.1	2.9
	ONAS1	16.1	11.7	4.2	9.7	8.2	5.8	4	5.1	2.3
	ONAS2	13.6	11.2	7.9	6.6	9.3	4.5	3	2.6	1.3
	ONAS3	21.1	17.8	9.7	13.3	11.2	8.1	10.6	8.1	3
	ONAS4	11.2	8.7	5.5	6	7.4	5.2	3.7	2.5	0.9
	ONAS5	17.1	15.6	8.9	11.6	10.5	5.5	5.6	5.3	2.3
	ONAS6	15.4	13	8.4	9.6	8.4	6.2	5.3	5.1	3.1
	ONAS7	18.2	12.8	7.8	9.3	11.3	6.2	3.9	4.7	sup
	QCAS1	5.7	3.7	0.8	3.3	2.9	1.8	2.8	2.6	1.4
	NBAS1	15.1	13	0	15.1	sup	sup	8.6	10.8	0
	NSAS1	19.9	17	sup	15.6	15.6	5.7	14.2	5.7	sup
	TRAS1	28.2	18.8	sup	sup	18.8	sup	sup	0	sup
**Space–time**	BCAST1	66.9	58.1	54.1	19.6	48.7	16.2	sup	3.4	sup
	BCAST2	49.4	43.3	41.3	10.8	33.2	14.9	2.7	sup	sup
	BCAST3	37.5	29.7	28.6	11	24.8	7.2	sup	2.2	sup
	ABAST1	24.5	22	18.7	11.2	17.7	8.9	7	4.9	1.9
	ONAST1	15	12.7	9.6	8.1	10.3	6.8	4	2.6	1.4
	ONAST2	15.5	12.7	6.5	9.7	7.5	5.6	4	5	2.4
	ONAST3	15.2	13.7	10.3	6.9	12.2	4.2	3	3.4	1.9
	QCAST1	11.5	8.6	sup	7.6	5.7	3.8	4.8	4.8	sup
	NSAST1	23.7	21.4	sup	19	21.4	sup	19	sup	sup

^a^Mortality rates are based on counts randomly rounded to base 3. Mortality rates are equal to the number of AATDs per 100,000 population per year. More than one substance type ccould be detected from a single AATD

## Discussion

This study presents the first national analysis using spatial scan statistics to identify clusters in space, time, and space–time of AATDs in Canada. Our results provide evidence for spatiotemporal heterogeneity in AATD rates at the national and P/T levels. This heterogeneity was also depicted by corresponding choropleth maps of AATD rates at the CD level across the country. Furthermore, this study provides evidence that detected substance types among AATDs differed substantially between clusters, pointing to the complexity of factors influencing the occurrence of AATDs in Canada. Results from these analyses in combination with more recent data can be used to guide interventions by targeting the regions highlighted in this study and the substance types most commonly detected, guide future research, and provide a basis for future comparisons.

At the national level, space and space–time analyses identified eight clusters in five regions where AATDs occurred at statistically significantly higher rates across Canada. These clusters highlighted western Canada (BC and western AB), northern ON/eastern MB, Manitoulin area, southeastern ON, and Oshawa, as regions that had substantially higher AATD rates when compared to the national mean (Figs. 2 and 3). The elevated rates of AATDs identified as clusters spatially aligned well with the AATD mortality rates depicted by the choropleth maps (Figs. 2, 3, 4 and 5). These regions have been highlighted by several other studies, including the National Report concerning ATD across Canada [12, 13, 27]. These clusters had risk ratios at least 2 to 3 times higher than regions outside of them (Tables 4 and 5). These risk ratios further highlighted the geographic disparity between regions within clusters compared to those outside of them. It is important to note that QC, PE, and NL had substantially lower AATD rates when compared to other Canadian P/Ts (Table 1). These provinces had substantially different substance profiles detected, where they had low rates of both fentanyl and non-fentanyl opioids, which may explain some of the reason for the relatively low rates observed (Table 1).

At the P/T level, space and space–time analyses identified 24 clusters in 15 regions where AATDs occurred at statistically significant higher rates across Canada. These clusters highlighted southern YT including Whitehorse, Greater Vancouver, southern BC including Kamloops, Kelowna, and many of the communities along the Fraser River, Abbotsford and its surrounding areas, Calgary and its surrounding areas, Edmonton to Red Deer, the areas just west of Calgary, northern ON including Thunder Bay, southeastern ON, Oshawa, Kingston, Barrie and the areas north of Barrie, northwestern and southwestern QB, Saint John NB, and Cape Breton NS, as regions that had substantially higher AATD rates when compared to their respective P/T mean (Figs. 4 and 5, Tables 4 and 5). These clusters had risk ratios at least 2 to 3 times higher than the regions outside of them (Tables 4 and 5). In the last two decades, some of these regions, for example, Vancouver, have been noted extensively in literature for having particularly high rates of drug-related outcomes [7, 14–18, 28, 29]. The clusters in of southern BC which included Kamloops, Kelowna, and many of the communities along the Fraser River identified in this study closely resembled a cluster identified by Hu et al., [14]. However, regions such as Saint John NB, Barrie, Cape Breton, and northern Ontario have rarely been highlighted in literature as having had high rates of AATD. This may suggest that despite having high rates of AATDs, some regions may be overlooked.

Purely temporal and space–time clusters generally occurred in 2017 (S. Figure 1). This is likely reflecting the general increase in AATDs over the study period. Interestingly, cluster ABAT had a very short time window where fentanyl was detected heavily in these AATDs (Tables 2 and 3). However, there were no space–time clusters specifically associated with November or December 2017 when it occurred. This may be because space–time methods were aggregated to the month due to computational limitations, while temporal analyses were measured to the day. Though, other methods, such as space–time permutation models, are better used for identifying local outbreak events [30]. No strong evidence of a seasonal trend in AATDs across Canada was observed. However, if a seasonal trend did exist, it may be overshadowed by the relative increase in AATDs throughout the study period and the short time frame examined.

As AATD usually involves more than one substance type [7, 13, 31], the detection of different substance types associated with AATDs within clusters was explored. In general, throughout all clusters, opioids were the most frequently detected substance type among AATDs (Tables 1, 3, 6 and 7). However, whether the most common opioids were fentanyl or non-fentanyl opioids varied by region. Where fentanyl was detected less, there were typically higher rates of non-fentanyl opioids, benzodiazepines, antidepressants, and antipsychotics detected. Alcohol and stimulant-related AATDs were relatively high across all provinces and clusters and tended to be the second and third most detected substance types after opioids, respectively (Tables 1, 6 and 7).

Fentanyl-related AATDs were highest in western Canada, where it had first been reported in Canada in 2011 [13], but gradually decreased among AATDs that occurred further east and often further north from the United States border, with fentanyl-related AATDs being quite rare in QC and the Atlantic provinces of eastern Canada (Tables 1, 6 and 7). These results may reflect the increasing presence of fentanyl/fentanyl-related analogues and their origin in the drug supply throughout this study period, such as carfentanil which was found in the highest proportions in BC and AB among all carfentanil-related seized samples tested by Health Canada’s Drug Analysis Service from 2016 to 2017 [13, 32, 33]. Additionally, both inside and outside of clusters, we found that benzodiazepines, antipsychotics, and antidepressants were commonly detected in AATDs across Canada, but benzodiazepines and antidepressants were detected particularly often relative to the total AATDs in the Maritime provinces and rarely in BC (Tables 1, 6 and 7). Although this may be due in part to the absence of information on BC AATDs that only involved prescribed substances or alcohol, as discussed in the methods. The distribution of detected substance type rates in AATDs forming clusters tended to follow a similar order to the substance types most detected overall in the P/Ts in which the clusters occurred (Tables 1, 6–7). However, within cluster rates were typically much higher for each of the substance types. Though there were exceptions, such as the clusters in northern ON, where the order of most detected substances differed substantially from that of ON overall (Tables 1, 6–7). This highlights that, although there were major regional differences in substance types between clusters, substance-specific interventions targeted at the provincial level may also broadly address the substance types most affecting regions within clusters. The most current estimates of opioids and stimulants contributing to AATDs showed stimulant and opioid AATDs peaked in 2021 and reduced slightly in 2022. When compared to detected opioid and stimulant AATDs in this study (Table 1), these estimates showed a general increase across P/Ts, with particularly high increases in opioid AATDs in BC, AB, SK, ON, QC, and particularly high increases in stimulant AATDs in SK, MB, and ON [7].

This study focuses on the "big picture" regarding the spatiotemporal distribution of AATDs in Canada. Similar to research by Hernandez et al., our study shows that a large portion of total AATDs are from regions disproportionately affected by drug-related harms, highlighting the importance of targeting interventions [36]. We recommend that future studies use space–time permutation models to identify clusters that are a result of major changes through time, that are not biased by purely spatial and purely temporal clusters [30]. Finding these non-endemic events using space–time permutation models, potentially like those associated with “bad batches” of drugs [32–35], may give insight into specific events that result in major changes in AATDs through time. Further studies are needed to gain deeper insight into the causes of these clusters. These studies should be repeated often to monitor changes in AATDs through time, including changes in the substances contributing to or involved in these deaths, and to measure the impact of any given intervention. Further studies should also examine why QC, PE, and NL had substantially lower AATD rates when compared to other Canadian P/Ts (Table 1). If the location of non-substance-related deaths were available, Bernoulli models could also be used to examine clusters of proportional morbidity. We also attempted to identify clusters in space, time, and space–time regarding those who died of suicide-related AT, however, there was inadequate statistical power using data only from this time period to report any comprehensive or meaningful results. This should be explored in the future using data from a longer time period. Lastly, it would be meaningful to use information on non-fatal overdoses to examine the probability of surviving an AT event to help further understand the efficacy of interventions used across Canada.

### Limitations

Several limitations should be considered when interpreting the results of this study. The data analysed in this study are at least six years old at the time of publication. More current publicly available data regarding all AATDs does not yet exist, however, more recent data concerning opioid-related AATDs showed that they have more than doubled from 2017 to 2022 [8]. This highlights that opioid-related AATDs have worsened substantially since the study period and the climate regarding AATDs changes quickly. Therefore, it is necessary to emphasize that the findings in this study are related to the 2016 and 2017 period and in order to understand more recent events, similar studies using more recent data need to be performed. The scanning window in the spatial cluster analysis was limited to a circular shape. This means for the analysis at the CSD level, CSD data are considered in the scanning window when the centroid of the CSD falls within the window. The resulting significant cluster is visualized on the map as a circle, centred by the centroid of the window location, with a radius of length to the furthest CSD centroid, for the CSDs included in the cluster. As a result, the mapped circle can overlap parts of CSDs that were not part of the significant cluster [37]. This approach to the spatial scan statistic works best when the spatial units used to aggregate data are uniform in area and shape. As the Canadian CSDs vary in area and shape, with smaller units in more populated areas. Therefore, the large clusters detected by this analysis should not be interpreted as indicating that the entire area has elevated AATD rates, but rather, should be interpreted as a broad-brush guide to highlight regions in the country where we would expect to find communities with elevated rates. Furthermore, geographic units were limited to CSDs, more discrete geographic units would allow for higher-resolution clusters. Our analyses used the smallest geographic unit possible. It should be noted that when using aggregate data, results may differ based on the level the researcher chooses to aggregate their data to (modifiable area unit problem) [38]. However, as the rates for CSDs and CDs did not differ considerably, and our clusters were typically large and included many CSDs, clusters may not differ substantially if we had information that allowed us to use unaggregated or aggregated to a higher resolution.

Data quality and availability limitations may have affected the results. Differences in substances tested, testing practices, and laboratory equipment across the P/Ts and over time, could have resulted in inaccurate prevalence estimates in this study. In particular, data from BC did not include AATDs if the cause of death was only from prescribed pharmaceutical substances or alcohol. Therefore, AATD rates from BC were likely underestimated and this error would likely differ by substance types detected. We felt choosing substance types by their detection in post-mortem examinations would give a broader perspective on the AATDs that occurred. This choice does not impact in any way the detection of clusters, but would impact the substance-type characterization of clusters. We chose to prioritize the location of the individual based on the CSD of residence rather than event or death locations. We assume the residential location is more representative of risk factors for AATD and more meaningful for policies this information may influence. Furthermore, the majority of AATDs occurred at the individual’s residence (65%) [7]. However, residence, event, and death locations at the CSD level were largely in agreement with each other, therefore focusing on either event or death location would likely not cause substantial changes to the clusters. It should be noted that some variation in clusters would still likely be present if event or death locations were prioritized instead of residence. Lastly, the data analysed took at least two years to collect and characterize a period six years prior to this study's publication date. Though the results can provide a comprehensive baseline to compare with more current data, current rates, clusters, and cluster information may differ substantially. Improvements in the data reporting process and the comparability of data across P/Ts could facilitate and enhance the efficiency and timeliness of similar studies in the future.

## Conclusion

Since the end of the study period, the rate of AATDs has continued to increase [2, 3]. It is of growing importance to understand how substances, both legal and part of the illegal drug supply, affect populations living in Canada, and how these dynamics change over time. In this study, we identified eight clusters in five regions at the national level as well as 24 clusters in 15 regions at the P/T level that highlight where AATD rates occurred far higher than the national or P/T mean. Information on the areas most impacted by AATDs can be used by institutions from the municipal to the federal level to help guide the targeting of interventions aimed at reducing substance-related harms. The descriptive statistics of each cluster should help highlight what drug types most impacted these clusters, helping to further target substance-specific interventions within these areas, as even within P/Ts, not all clusters observed the same patterns of substances that were commonly detected among AATDs. It is imperative that such studies continue and are updated frequently to understand how AATDs change with time, which substances are affecting which regions, and to measure the impact of any given intervention. We hope this study assists in directing future research to identify factors that contribute to these clusters and provides a baseline for future comparison, to help reduce the harms caused by these substances.

### Supplementary Information

Supplementary Material 1.

Supplementary Material 2.

## Data Availability

The national chart review of coroner and medical examiner files dataset analyzed in this study is not publicly available due to provisions in the data use agreements with provincial and territorial data providers. For inquiries regarding access to this dataset, interested researchers may contact: Jenny Rotondo. Senior epidemiologist. Public Health Agency of Canada. jenny.rotondo@phac-aspc.gc.ca.
