# Supplementary figures and images for: Spatiotemporal epidemiology of substance-related accidental acute toxicity deaths in Canada from 2016 to 2017
Source: BMC Public Health. 2024 Jun 20;24:1641. doi: 10.1186/s12889-024-18883-2 (PMC11188508; doi:10.1186/s12889-024-18883-2)

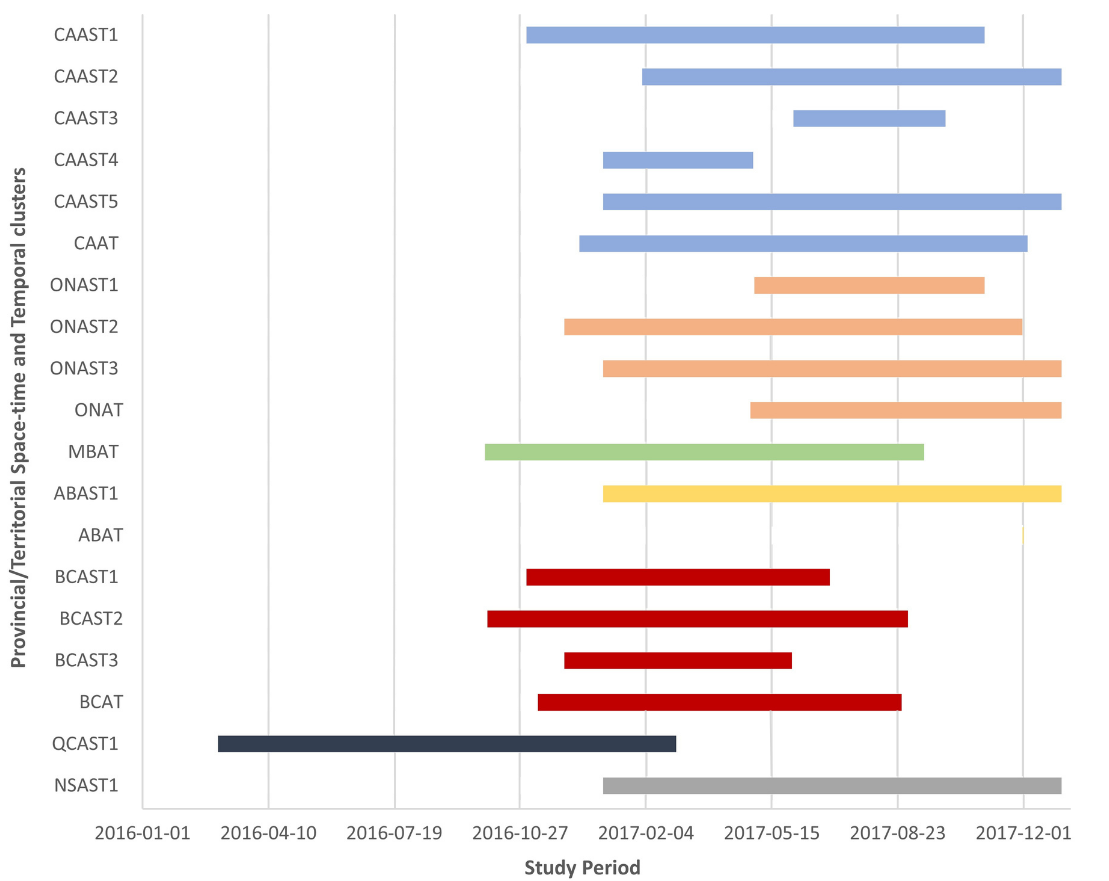

Supplement: Supplementary file 1 — Supplementary Material 1. [file 12889_2024_18883_MOESM1_ESM.png]
