# Supplementary material for: Spatiotemporal epidemiology of substance-related accidental acute toxicity deaths in Canada from 2016 to 2017
Source: BMC Public Health. 2024 Jun 20;24:1641. doi: 10.1186/s12889-024-18883-2 (PMC11188508; doi:10.1186/s12889-024-18883-2)
Supplement: Supplementary file 2 — Supplementary Material 2. [file 12889_2024_18883_MOESM2_ESM.docx]

Supplementary table 1. Substances included in each substance type category.

| Substance type | Substances |
| --- | --- |
| Alcohol | Ethanol, isopropanol, methanol |
| Antipsychotics | Aripiprazole, asenapine, chlorpromazine, clozapine, flupenthixol, fluphenazine, haloperidol, levomepromazine, loxapine, lurasidone, olanzapine, paliperidone, pimozide, prochlorperazine, quetiapine, risperidone, ziprasidone, zuclopenthixol |
| Benzodiazepines | Alprazolam, bromazepam, chlordiazepoxide, clobazam, clonazepam, diazepam, etizolam, flubromazolam, flurazepam, lorazepam, midazolam, nitrazepam, oxazepam, temazepam, triazolam, unspecified benzodiazepines |
| Fentanyl opioids | 3-methylfentanyl, 4-fluorobutyrfentanyl, 4-fluoroisobutyryl fentanyl, acetylfentanyl, acrylfentanyl, butyrylfentanyl, carfentanil, cyclopropyl/crotonyl fentanyl, despropionyl-fentanyl, fentanyl, furanylfentanyl, methoxyacetylfentanyl, remifentanil, sufentanil |
| Non-fentanyl opioids | Buprenorphine, codeine, dextrorphan, diacetylmorphine (heroin), dihydrocodeine, embutramide, hydrocodone, hydromorphone, meperidine, methadone, mitragynine, morphine, oxycodone, oxymorphone, pentazocine, propoxyphene, tapentadol, thebaine, tramadol, U-47700, U-49900, U-51754 |
| Opioids | People who died with any of the fentanyl or non-fentanyl opioids described above as detected or a cause of death in the coroner or medical examiner file. |
| Stimulants | 1,3-trifluoromethylphenylpiperazine (TFMPP), 2,5-dimethoxy-4-bromo-amphetamine, 3-fluorophenmetrazine, 4-fluoroamphetamine, alpha-pyrrolidinovalerophenone, aminorex, amphetamine, beta-phenethylamine, butylone, caffeine, cocaine, dextroamphetamine, ethylone, ethylphenidate, lisdexamfetamine, methamphetamine, methylenedioxyamphetamine (MDA), methylenedioxymethamphetamine (MDMA), methylphenidate, N-benzylpiperazine (BZP), nicotine, paramethoxyamphetamine (PMA), paramethoxymethamphetamine (PMMA), phentermine, theobromine, unspecified stimulants |
| Antidepressants | Amitriptyline, amoxapine, bupropion, citalopram, clomipramine, desipramine, desvenlafaxine, doxepin, duloxetine, fluoxetine, fluvoxamine, imipramine, mirtazapine, moclobemide, nortriptyline, paroxetine, sertraline, trazodone, trimipramine, venlafaxine, vortioxetine unspecified tricyclic antidepressants |

Supplementary table 2. Accidental acute toxicity death (AATD) rate ranges across census divisions in Canada, 2016 to 2017.

| Province | CDUID | Census division | AATD rate range ^a^ |
| --- | --- | --- | --- |
| Quebec | 2464 | Les Moulins | > 0 to 10 |
| Ontario | 3519 | York | > 0 to 10 |
| Quebec | 2465 | Laval | > 0 to 10 |
| Quebec | 2473 | Therese-De Blainville | >1 to 10 |
| Quebec | 2458 | Longueuil | >1 to 10 |
| Quebec | 2471 | Vaudreuil-Soulanges | >1 to 10 |
| Quebec | 2466 | Montreal | >1 to 10 |
| Quebec | 2423 | Quebec | >1 to 10 |
| Quebec | 2443 | Sherbrooke | >1 to 10 |
| Quebec | 2494 | Le Saguenay-et-son-Fjord | >1 to 10 |
| Quebec | 2449 | Drummond | >1 to 10 |
| Ontario | 3538 | Lambton | >1 to 10 |
| Ontario | 3524 | Halton | >1 to 10 |
| Newfoundland | 1001 | Division No. 1 | >1 to 10 |
| Ontario | 3521 | Peel | >1 to 10 |
| Ontario | 3547 | Renfrew | >1 to 10 |
| Quebec | 2475 | La Riviere-du-Nord | >1 to 10 |
| Quebec | 2437 | Francheville | >1 to 10 |
| Ontario | 3536 | Chatham-Kent | >1 to 10 |
| Nova Scotia | 1209 | Halifax | >1 to 10 |
| New Brunswick | 1307 | Westmorland | >1 to 10 |
| Ontario | 3506 | Ottawa | >1 to 10 |
| Ontario | 3523 | Wellington | >1 to 10 |
| Ontario | 3532 | Oxford | >1 to 10 |
| Manitoba | 4607 | Division No. 7 | >1 to 10 |
| Ontario | 3542 | Grey | >1 to 10 |
| Quebec | 2481 | Gatineau | >1 to 10 |
| Ontario | 3539 | Middlesex | >1 to 10 |
| Ontario | 3512 | Hastings | >1 to 10 |
| Saskatchewan | 4711 | Division No. 11 | >1 to 10 |
| Ontario | 3514 | Northumberland | >1 to 10 |
| Ontario | 3518 | Durham | >1 to 10 |
| Ontario | 3528 | Haldimand-Norfolk | >1 to 10 |
| Ontario | 3537 | Essex | >1 to 10 |
| Ontario | 3544 | Muskoka | >1 to 10 |
| Ontario | 3556 | Cochrane | >1 to 10 |
| Alberta | 4810 | Division No. 10 | >10 to 20 |
| Alberta | 4812 | Division No. 12 | >10 to 20 |
| Alberta | 4813 | Division No. 13 | >10 to 20 |
| Ontario | 3548 | Nipissing | >10 to 20 |
| Ontario | 3520 | Toronto | >10 to 20 |
| Saskatchewan | 4715 | Division No. 15 | >10 to 20 |
| Saskatchewan | 4706 | Division No. 6 | >10 to 20 |
| Ontario | 3543 | Simcoe | >10 to 20 |
| Ontario | 3515 | Peterborough | >10 to 20 |
| Manitoba | 4611 | Division No. 11 | >10 to 20 |
| Ontario | 3529 | Brant | >10 to 20 |
| Ontario | 3530 | Waterloo | >10 to 20 |
| Ontario | 3525 | Hamilton | >10 to 20 |
| Alberta | 4801 | Division No. 1 | >10 to 20 |
| Alberta | 4802 | Division No. 2 | >10 to 20 |
| British Columbia | 5903 | Central Kootenay | >10 to 20 |
| Ontario | 3526 | Niagara | >10 to 20 |
| Alberta | 4817 | Division No. 17 | >10 to 20 |
| British Columbia | 5901 | East Kootenay | >10 to 20 |
| Manitoba | 4622 | Division No. 22 | >10 to 20 |
| Ontario | 3553 | Greater Sudbury / Grand Sudbury | >10 to 20 |
| New Brunswick | 1301 | Saint John | >10 to 20 |
| British Columbia | 5931 | Squamish-Lillooet | >10 to 20 |
| British Columbia | 5929 | Sunshine Coast | >10 to 20 |
| Alberta | 4803 | Division No. 3 | >10 to 20 |
| Ontario | 3510 | Frontenac | >10 to 20 |
| British Columbia | 5939 | Columbia-Shuswap | >10 to 20 |
| Ontario | 3557 | Algoma | >10 to 20 |
| Ontario | 3560 | Kenora | >10 to 20 |
| Alberta | 4805 | Division No. 5 | >10 to 20 |
| Alberta | 4811 | Division No. 11 | >10 to 20 |
| Alberta | 4806 | Division No. 6 | >10 to 20 |
| British Columbia | 5926 | Comox Valley | >10 to 20 |
| Saskatchewan | 4716 | Division No. 16 | >10 to 20 |
| Nova Scotia | 1217 | Cape Breton | >10 to 20 |
| British Columbia | 5941 | Cariboo | >20 to 30 |
| Alberta | 4816 | Division No. 16 | >20 to 30 |
| British Columbia | 5953 | Fraser-Fort George | >20 to 30 |
| British Columbia | 5919 | Cowichan Valley | >20 to 30 |
| British Columbia | 5905 | Kootenay Boundary | >20 to 30 |
| Ontario | 3558 | Thunder Bay | >20 to 30 |
| British Columbia | 5917 | Capital | >20 to 30 |
| British Columbia | 5907 | Okanagan-Similkameen | >20 to 30 |
| Alberta | 4808 | Division No. 8 | >20 to 30 |
| Yukon | 6001 | Yukon | >20 to 30 |
| British Columbia | 5937 | North Okanagan | >20 to 30 |
| Alberta | 4815 | Division No. 15 | >20 to 30 |
| British Columbia | 5923 | Alberni-Clayoquot | >20 to 30 |
| British Columbia | 5915 | Greater Vancouver | >20 to 30 |
| Alberta | 4819 | Division No. 19 | >20 to 30 |
| British Columbia | 5927 | Powell River | >20 to 30 |
| British Columbia | 5955 | Peace River | >20 to 30 |
| British Columbia | 5909 | Fraser Valley | >20 to 30 |
| British Columbia | 5921 | Nanaimo | >30 |
| British Columbia | 5924 | Strathcona | >30 |
| British Columbia | 5935 | Central Okanagan | >30 |
| British Columbia | 5933 | Thompson-Nicola | >30 |
| Manitoba | 4619 | Division No. 19 | >30 |

Notes: ^a^ Suppressed census divisions (n=163) and those with no death (n=36) are not presented in this table. Rate ranges are presented as deaths per 100,000 population.

Supplementary Table 3. Census subdivisions included in each accidental acute toxicity death cluster defined by numerical identifier*, Canada, 2016 to 2017.

| Cluster | Included Census subdivisions |
| --- | --- |
| CAAS1 | 5924030, 5924029, 5924836, 5924048, 5924833, 5924805, 5924814, 5943033, 5924835, 5924025, 5924803, 5924806, 5924813, 5923806, 5923824, 5924039, 5943807, 5924042, 5943835, 5943008, 5943837, 5943017, 5943012, 5923808, 5943031, 5943808, 5923049, 5924034, 5924812, 5923813, 5943817, 5943816, 5924840, 5924804, 5943804, 5943815, 5943023, 5926024, 5923825, 5923025, 5924817, 5943806, 5943809, 5923823, 5943027, 5926014, 5926802, 5943813, 5924052, 5923035, 5926010, 5926022, 5926801, 5924820, 5924818, 5926005, 5923804, 5926021, 5923019, 5923807, 5923810, 5923033, 5943836, 5943037, 5923822, 5923037, 5927802, 5923816, 5923801, 5921036, 5927008, 5924054, 5923008, 5923805, 5923802, 5927012, 5921806, 5923039, 5923814, 5927018, 5923809, 5923803, 5927806, 5923047, 5927016, 5921023, 5921032, 5921034, 5927010, 5927020, 5921018, 5919814, 5921030, 5921016, 5929018, 5921805, 5919035, 5945803, 5921008, 5929803, 5921007, 5919033, 5929011, 5921804, 5929022, 5921807, 5919015, 5917815, 5929024, 5921010, 5919812, 5919017, 5921014, 5919016, 5919816, 5929026, 5919804, 5919021, 5929005, 5929028, 5929801, 5919811, 5919821, 5919802, 5919809, 5919817, 5917056, 5919051, 5945006, 5919801, 5941828, 5917805, 5915062, 5919803, 5941829, 5919008, 5931021, 5941876, 5931801, 5919012, 5941884, 5931808, 5915065, 5919822, 5931809, 5931806, 5931802, 5931807, 5931006, 5919818, 5919013, 5919046, 5915055, 5919808, 5941808, 5931017, 5919820, 5917027, 5915803, 5919049, 5931020, 5915808, 5941831, 5915807, 5915022, 5915051, 5919043, 5919815, 5915015, 5945010, 5945801, 5915046, 5915811, 5917029, 5917802, 5917054, 5915810, 5917005, 5945012, 5945802, 5917801, 5915806, 5917819, 5917052, 5917010, 5915802, 5917804, 5915025, 5917049, 5931012, 5917015, 5915011, 5915020, 5915036, 5945014, 5941039, 5917803, 5917044, 5915029, 5915043, 5915038, 5917042, 5917047, 5917809, 5931032, 5917041, 5941851, 5917021, 5917811, 5917812, 5931843, 5917040, 5915805, 5915034, 5915804, 5931812, 5915039, 5941871, 5917034, 5915004, 5917030, 5909875, 5941854, 5915007, 5909852, 5915070, 5941880, 5915809, 5915830, 5915801, 5941818, 5941840, 5915813, 5941845, 5909883, 5941817, 5909060, 5915002, 5909845, 5931844, 5915816, 5915075, 5941834, 5941858, 5915001, 5915835, 5941839, 5941838, 5941879, 5915840, 5941848, 5941827, 5941844, 5909848, 5915825, 5909056, 5931826, 5941859, 5909842, 5931833, 5909878, 5949802, 5909048, 5931831, 5909035, 5909052, 5941856, 5941841, 5931034, 5941041, 5909877, 5909062, 5941821, 5941860, 5909881, 5909880, 5941883, 5931824, 5931813, 5909879, 5909882, 5941861, 5931821, 5931814, 5909826, 5931828, 5931026, 5909835, 5909885, 5909824, 5931823, 5909821, 5909825, 5931819, 5931818, 5909833, 5909839, 5931832, 5909849, 5931842, 5909884, 5909831, 5909830, 5909827, 5909020, 5931816, 5931822, 5931827, 5931815, 5931820, 5933819, 5931817, 5941862, 5909014, 5933858, 5933839, 5933840, 5931829, 5933848, 5909838, 5909027, 5933874, 5933878, 5909032, 5941804, 5933870, 5933841, 5933868, 5933873, 5941863, 5933814, 5933842, 5933813, 5909837, 5909834, 5933852, 5933833, 5933866, 5933855, 5933850, 5933853, 5909832, 5933865, 5933824, 5933879, 5941806, 5933825, 5933829, 5933849, 5933832, 5933823, 5933015, 5933831, 5933882, 5909844, 5933896, 5933897, 5933821, 5909843, 5941850, 5933803, 5933872, 5909805, 5941849, 5909810, 5909850, 5909807, 5933037, 5909814, 5909819, 5909034, 5909815, 5941807, 5909806, 5909847, 5909804, 5909036, 5933875, 5941801, 5909817, 5909836, 5933851, 5909816, 5909818, 5909812, 5909876, 5909809, 5909009, 5909808, 5909841, 5941811, 5933836, 5933895, 5941012, 5933032, 5941873, 5933881, 5933837, 5933028, 5941833, 5933810, 5941868, 5941872, 5933834, 5941885, 5933846, 5933828, 5933019, 5933854, 5933812, 5933857, 5933861, 5933844, 5933845, 5941864, 5933024, 5933876, 5933859, 5941835, 5909016, 5941881, 5941837, 5949020, 5933808, 5941027, 5941009, 5933035, 5951031, 5941810, 5941812, 5933809, 5933012, 5941805, 5941015, 5941010, 5941882, 5933801, 5941855, 5933807, 5933817, 5941803, 5933006, 5941005, 5933805, 5941852, 5941866, 5933811, 5947806, 5933039, 5941019, 5941813, 5907055, 5941017, 5933806, 5941809, 5907024, 5941013, 5941802, 5951818, 5951826, 5951825, 5951833, 5951824, 5951017, 5933008, 5941021, 5951819, 5933802, 5951823, 5951822, 5933042, 5933880, 5933877, 5941016, 5949803, 5951019, 5949005, 5941014, 5951807, 5947032, 5951813, 5951009, 5933044, 5951806, 5907807, 5947021, 5951809, 5953042, 5907053, 5933888, 5933074, 5951844, 5951022, 5933887, 5951802, 5951815, 5951827, 5907809, 5933886, 5933060, 5907051, 5951007, 5953046, 5933045, 5907808, 5951820, 5935018, 5941025, 5935020, 5951034, 5907009, 5933898, 5951028, 5907035, 5941026, 5933884, 5947807, 5951821, 5933889, 5907803, 5933054, 5933072, 5935029, 5933067, 5935802, 5907806, 5907801, 5907026, 5953044, 5935803, 5939802, 5907805, 5939805, 5907041, 5939801, 5937017, 5951847, 5907049, 5907047, 5935010, 5953023, 5939039, 5935016, 5907014, 5935801, 5939807, 5953038, 5937801, 5937014, 5937803, 5951804, 5949011, 5939804, 5907802, 5947804, 5949807, 5907028, 5939806, 5949018, 5939808, 5951803, 5933068, 5947026, 5907005, 5939811, 5907022, 5939803, 5951849, 5939032, 5949804, 5951038, 5949805, 5937805, 5951801, 5951013, 5949013, 5953801, 5947007, 5951840, 5937024, 5939037, 5937028, 5935012, 5937010, 5949844, 5951051, 5951830, 5951805, 5937802, 5937033, 5937021, 5951043, 5951810, 5947012, 5951829, 5937005, 5939045, 5947016, 5939044, 5937022, 5947810, 5905054, 5951053, 5951032, 5951814, 5947030, 5937041, 5947809, 5949817, 5949819, 5949820, 5951846, 5949810, 5933070, 5951845, 5953048, 5939043, 5905037, 5947027, 5949814, 5905042, 5949816, 5953012, 5937023, 5947023, 5905052, 5947803, 5949024, 5953019, 5949811, 5951015, 5949022, 5949812, 5951828, 5905032, 5951811, 5949815, 5949818, 5953007, 5953050, 5949035, 5949813, 5949028, 5939019, 5953802, 5905050, 5939023, 5951848, 5903060, 5903058, 5903050, 5905030, 5905023, 5953033, 5905018, 5903045, 5905014, 5903052, 5903056, 5903019, 4814028, 5905009, 5905026, 5905005, 5903032, 5903027, 5903015, 5903043, 4815033, 4818005, 5903011, 5939011, 5955003, 5903047, 4815037, 5903041, 5903039, 5903023, 5939007, 5949039, 5955023, 5955021, 5903010, 5903017, 5955010, 5955802, 5949032, 4814019, 5903807, 5903004, 5955801, 5955025, 5901048, 5903013, 5901040, 5901039, 5901806, 5901037, 4815032, 4819815, 4819009, 5901028, 5901804, 4819008, 4819011, 5955812, 5901043, 4809810, 5901046, 4819006, 5955014, 5955005, 4818015, 5901022, 5901803, 4819012, 4814003, 4815035, 5901802, 5955808, 5901035, 5901805, 5955030, 5955034, 4819014, 5955042, 4815023, 4809002, 4814024, 4819059, 5955040, 5901019, 4815015, 5901012, 4818002, 4819058, 5955807, 5955803, 4819056, 4818816, 5901801, 4815013, 4819054, 5901003, 4815802, 4809809, 5955804, 4809806, 4815030, 4818818, 5901006, 4815027, 5901017, 4818018, 4809005, 4809015, 4809010, 4806036, 4819049, 4806019, 4815007, 4811032, 4806803, 4819066, 4806031, 5949041, 4806804, 4819068, 4815045, 4806009, 4817064, 4813030, 4811031, 4806008, 4806011, 4806028, 4806001, 4808024, 4819041, 4806016, 4806014, 4819048, 4806032, 4817825, 4806029, 4806034, 4806021, 4806026, 4819826, 4803016, 4806012, 4803011, 4819046, 4813002, 5949832, 4819044, 4808027, 4808025, 4819071, 4813029, 4806006, 4808006, 4817062, 4811026, 4819072, 4808013, 4808044, 4811038, 4803014, 4808023, 4808012, 4806017, 4811039, 4808005, 4819042, 4817021, 4803026, 4808008, 4819074, 4813010, 4813015, 4808026, 4803003, 4808042, 4811024, 4808009, 4813001, 4811041, 4808028, 4803024, 4811045, 4803018, 4811042, 4817024, 4811044, 4808001, 4811034, 4811806, 4817039, 4811807, 4806022, 4811005, 4803022, 4808011, 4803801, 4813006, 4813811, 4811004, 4811020, 5949843, 4808038, 4817829, 4811006, 5949847, 4817027, 4811022, 4808029, 4819038, 4811007, 4806024, 4811023, 4803803, 4811003, 4813005, 4811001, 4803021, 4803008, 4813008, 4805044, 4817830, 4811009, 4811008, 4813012, 4808022, 4813007, 4817836, 5949846, 4811802, 4813011, 4808031, 4805046, 4813009, 4811021, 5959810, 4805018, 4803006, 4811046, 4817828, 4803019, 4813013, 4813018, 4813014, 4805006, 4808039, 4811048, 4813019, 4813003, 4811012, 4805041, 4805048, 4811019, 4805011, 4808032, 4805004, 4811803, 4805022, 4805049, 4811049, 4813017, 4803004, 4805042, 4813016, 4805002, 4803802, 4811018, 4808813, 4808002, 4811801, 4817831, 4808812, 4802016, 4808004, 4811805, 4811804, 4802014, 4808811, 4805021, 4805001, 4805012, 5949845, 4811011, 4808034, 4805802, 4811016, 5949038, 4811002, 4803001, 4805009, 4817078, 4802013, 5959805, 4811062, 4811013, 4811061, 4802012 |
| CAAS2 | 3560098, 3560085, 3560090, 3560100, 3560075, 3560088, 3560086, 3560059, 3560096, 3560095, 3560097, 3560093, 3560081, 3560079, 3560049, 3560076, 3560104, 3560055, 3560054, 3560053, 3560102, 3558085, 3560080, 3560071, 3560046, 4622056, 3560070, 3558080, 3558097, 3560052, 3560077, 3560067, 4622048, 4622055, 3560042, 4622801, 4623071, 4622800, 3560056, 3560032, 4622049, 3560078, 3558065, 3560091, 3560034, 3558076, 3560084, 3558090, 4619079, 4619051, 3558077, 3558075, 3560027, 4622050, 3558069, 3560001, 3560057, 3558068, 3560024, 3560058, 3560021, 3558067, 3558095, 4622046, 3558064, 3558044, 3558100, 3558041, 3560061, 4623022, 3560089, 3558034, 3560010, 4623027, 4619052, 3560066, 4619053, 4623025, 4619077, 3558063, 3559001, 3560004, 3558028, 3560069, 3560051, 3560005, 3560083, 4619075, 4622802, 4622064, 3558051, 3558019, 4618091, 3560008, 4619054, 3558054, 3559090, 3559066, 3558004, 3560063, 3558066, 3558011, 4601071, 4601075, 4619050, 3560007, 3558016, 3559048, 4619061, 4619045, 3559064, 3560068, 3558003 |
| CAAS3 | 3518013 |
| ABAS1 | 4815013, 4806009, 4806803, 4806011, 4806804, 4806008, 4815802 |
| ABAS2 | 4811801, 4811803, 4808811, 4808812, 4811002, 4808813, 4808039, 4811011, 4810012, 4811802, 4811008, 4811001, 4808038, 4810004, 4811007, 4811009, 4811006, 4811004, 4811003, 4808032, 4811023, 4811005, 4811016, 4811022, 4810011, 4810014, 4810002, 4811012, 4808031, 4810006, 4811020, 4810001, 4808042, 4811019, 4808022, 4811013, 4808028, 4811021, 4808034, 4811018, 4808044, 4808029, 4808026, 4807028, 4807027, 4811024, 4810009, 4808011, 4807029, 4810003, 4811804, 4808023, 4808005, 4808027, 4811026, 4811061, 4808013, 4808025, 4808012, 4808004, 4810018, 4807026, 4811049, 4811048 |
| BCAS1 | 5915022, 5915803, 5915807, 5915808 |
| BCAS2 | 5933805, 5933006, 5933801, 5933811, 5933807, 5933809, 5933012, 5933808, 5933806, 5933008, 5933802, 5933859, 5933861, 5933035, 5933851, 5909847, 5909819, 5909815, 5909850, 5933834, 5909805, 5933803, 5909806, 5933881, 5933895, 5909817, 5933897, 5909836, 5933896, 5933857, 5933882, 5933810, 5933849, 5933879, 5933865, 5933823, 5933825, 5933824, 5933015, 5933829, 5933866, 5933832, 5933850, 5933831, 5933855, 5933853, 5933852, 5933833, 5933842, 5933813, 5933873, 5909816, 5933037, 5933841, 5909809, 5933846, 5933868, 5909014, 5933870, 5933854, 5907055, 5933878, 5933828, 5907024, 5933874, 5933876, 5933837, 5933042, 5935020, 5909818, 5933840, 5933848, 5933839, 5933019, 5933880, 5909876, 5933039, 5933844, 5933060, 5935018, 5933845, 5933817, 5907051, 5933858, 5909841, 5935029, 5909808, 5935802, 5933024, 5931827, 5909016, 5935803, 5907035, 5909812, 5937017, 5909009, 5933812, 5931832, 5931815, 5907807, 5935016, 5935010, 5935801, 5909804, 5933875, 5933044, 5931820, 5909814, 5937014, 5933836, 5937803, 5937801, 5907049, 5931823, 5931822, 5931828, 5909810, 5907803, 5933872, 5909807, 5933898, 5931026, 5931814, 5933877, 5931816, 5907053, 5931842, 5939039, 5931819, 5933045, 5909842, 5931821, 5909048, 5939804, 5907041, 5931817, 5931824, 5907809, 5933884, 5909843, 5909848, 5931818, 5939806, 5933054, 5937805, 5935012, 5937010, 5909832, 5907808, 5909027, 5909034, 5909032 |
| ONAS1 | 3558003, 3558004, 3558011, 3558001, 3558012, 3558016, 3558019, 3558028, 3558034, 3558041, 3558044, 3558100, 3558064, 3558063, 3558095, 3558069, 3558051, 3558065, 3558054, 3558090, 3559001, 3559060, 3558077, 3560001, 3558075, 3558059, 3559090, 3558062, 3558097, 3559066, 3558067, 3558068, 3558080, 3559065, 3558066, 3558061, 3558076, 3558060, 3559064, 3560034, 3560057, 3557091, 3560027, 3559062, 3559061, 3560056, 3559012, 3559063, 3558085, 3559011, 3560024, 3559068, 3559016, 3557078, 3560055, 3560021, 3559019, 3559069, 3557076, 3559026, 3557096, 3557079, 3559024, 3560063, 3560046, 3560049, 3560004, 3560005, 3560083, 3560007, 3559031, 3560084, 3559048, 3560008, 3559051, 3559052, 3559053, 3560053, 3559040, 3559047, 3559042, 3557095, 3560032, 3557077, 3560058, 3557066, 3560066, 3556095, 3560010, 3557061, 3560069, 3560089, 3560054, 3557075, 3560065, 3560060, 3556076, 3557074, 3552092, 3560093, 3552053, 3552058, 3560052, 3560042, 3552054, 3560081, 3557011, 3557051, 3560068, 3560064, 3560082, 3557014, 3557008, 3557016, 3560061, 3556077, 3557006, 3557001, 3557019, 3557004, 3557021, 3556073, 3557028, 3557026, 3560102, 3557035, 3560086, 3556070, 3560085, 3560077, 3556066, 3551034, 3560090, 3557038, 3560098, 3557073, 3552093, 3560079, 3556056, 3560080, 3560059, 3556100, 3556092, 3557041, 3556052, 3551094, 3551100, 3557040, 3560067, 3551044, 3557072, 3560100, 3557039, 3556048, 3560104, 3552052, 3551027, 3551026, 3551028, 3557071, 3560070, 3556027, 3560097, 3560096, 3557094, 3552023, 3551021, 3560071, 3551045, 3552028, 3551006, 3551041, 3556042, 3552026, 3560075, 3560088, 3552031, 3551017, 3551042, 3551040, 3556102, 3551001, 3551011, 3560095, 3556031, 3554056, 3556091, 3551043, 3554057, 3552051, 3553005, 3560076, 3556014, 3553040, 3554094, 3554042, 3552036, 3556098, 3554068, 3556033, 3552013, 3554054, 3554044 |
| ONAS2 | 3529005, 3529006, 3529020, 3530004, 3532045, 3529021, 3528037, 3532002, 3528035, 3530010, 3532042, 3525005, 3530013 |
| ONAS3 | 3518013 |
| ONAS4 | 3520005 |
| ONAS5 | 3526037, 3526053, 3526032, 3526028, 3526043 |
| ONAS6 | 3543071, 3543074, 3543072, 3543068, 3543015, 3543009, 3543023, 3543070, 3543064, 3544065, 3544071, 3543069, 3543052, 3543050, 3543042, 3544002 |
| ONAS7 | 3510010 |
| QCAS1 | 2499035, 2499806, 2499045, 2499810, 2499808, 2499040, 2499015, 2499050, 2499812, 2499010, 2487105, 2487115, 2499802, 2487120, 2487110, 2487100, 2487904, 2488904, 2487095, 2499814, 2499055, 2487080, 2488070, 2487085, 2487090, 2499060, 2488065, 2499005, 2487058, 2487050, 2487070, 2487075, 2488902, 2487025, 2487035, 2488080, 2488060, 2487042, 2488075, 2487020, 2488015, 2487030, 2487015, 2488055, 2488802, 2488010, 2488085, 2487010, 2487005, 2488035, 2488050, 2487902, 2488005, 2488040, 2499818, 2488090, 2499020, 2488022, 2488045, 2488030, 2489050, 2489045, 2486042, 2489015, 2489010, 2499030, 2499025, 2499804, 2489040, 2489804, 2489008, 2485105, 2485100, 2489912, 2490804, 2489902, 2485095, 2485806, 2489908, 2485075, 2485070, 2485804, 2485090, 2485080, 2485085, 2485045, 2485050, 2485055, 2485060, 2485065, 2489802, 2485037, 2489910, 2485030, 2485025, 2499070, 2492904, 2485020, 2499816, 2499075, 2485015, 2483912, 2491902, 2483908, 2483906, 2483804, 2485905, 2485803, 2485907, 2490012, 2485802, 2485010, 2479910, 2492045, 2479906, 2485005, 2492055, 2483902, 2492050, 2479912, 2492060, 2492902, 2462919, 2483904, 2479926, 2490802, 2491050, 2492040, 2479904, 2462918, 2492030, 2484902, 2492065, 2479914, 2492070, 2479922, 2491042, 2462922, 2492022, 2479115, 2462920, 2491035, 2479097, 2492015, 2479902, 2483095, 2462802, 2491030, 2491802, 2479110, 2483088, 2492005, 2492010, 2484100, 2493080, 2491025, 2479105, 2483085, 2493075, 2499877, 2483090, 2479088, 2479065, 2491015, 2483075, 2491020, 2493070, 2479916, 2494930, 2483802, 2483065, 2479078, 2484095, 2493005, 2493035, 2493065, 2483070, 2462908, 2491010, 2493042, 2491005, 2483060, 2479060, 2479022, 2493012, 2493060, 2462904, 2435906, 2493055, 2483055, 2479025, 2483040, 2484070, 2483045, 2493045, 2479050, 2479037, 2479920, 2479010, 2493030, 2484090, 2483050, 2493025, 2435904, 2462906, 2479015, 2462916, 2493020, 2484065, 2494250, 2462910, 2479030, 2499878, 2499080, 2494265, 2490017, 2494260, 2493908, 2494255, 2483032, 2484082, 2435908, 2479924, 2484055, 2490027, 2479047, 2462085, 2479005, 2484060, 2494245, 2483015, 2462902, 2484050, 2493904, 2462914, 2483020, 2493906, 2478130, 2494240, 2478120, 2484040, 2484035, 2478127, 2478095, 2494068, 2484030, 2480135, 2484045, 2483010, 2480145, 2462912, 2478115, 2483005, 2494928, 2462080, 2484025, 2462060, 2480140, 2434906, 2478102, 2493902, 2435055, 2480130, 2478100, 2484015, 2478070, 2484020, 2480090, 2482035, 2478075, 2435902, 2494235, 2484010, 2480095, 2451065, 2482010, 2422902, 2480125, 2478015, 2462055, 2478047, 2480103, 2480085, 2478065, 2484005, 2435045, 2478020, 2482015, 2480110, 2478042, 2434904, 2462070, 2478060, 2482030, 2478050, 2480115, 2478802, 2462065, 2478032, 2436033, 2452095, 2462075, 2480078, 2494230, 2451070, 2435010, 2478055, 2434902, 2435050, 2480020, 2434135, 2482020, 2462053, 2494220, 2435040, 2478010, 2480065, 2494225, 2482005, 2482025, 2480070, 2480027, 2477065, 2477012, 2476065, 2462047, 2477055, 2478005, 2462020, 2451075, 2421904, 2435005, 2477011, 2462025, 2452080, 2462015, 2480015, 2435027, 2452090, 2435035, 2480060, 2480055, 2452085, 2477022, 2480050, 2481017, 2451085, 2477060, 2451060, 2477050, 2480037, 2452075, 2480045, 2480010, 2435015, 2451050, 2462030, 2451055, 2494926, 2494215, 2451080, 2416902, 2462037, 2435020, 2461050, 2477043, 2434090, 2477030, 2476052, 2480005, 2462007, 2476035, 2475045, 2451040, 2463055, 2434115, 2434128, 2495902, 2452070, 2451025, 2477035, 2476030, 2461040, 2437240, 2451045, 2475040, 2451035, 2451030, 2437245, 2437235, 2451090, 2461045, 2463060, 2452055, 2452062, 2476025, 2434097, 2434085, 2434105, 2463065, 2476055, 2461035, 2452030, 2437250, 2476043, 2461030, 2475017, 2437230, 2494210, 2461020, 2451015, 2475028, 2422025, 2451020, 2451008, 2434048, 2475005, 2434078, 2463013, 2461025, 2463030, 2437215, 2476020, 2463048, 2461013, 2437225, 2437205, 2422035, 2452040, 2434060, 2496902, 2437067 |
| NBAS1 | 1301006 |
| NSAS1 | 1217008, 1217030, 1217020, 1218001, 1216011 |
| TRAS1 | 6001006, 6001007, 6001057, 6001047, 6001036, 6001046, 6001058, 6001008, 6001048, 6001044, 6001060, 6001009, 6001010, 6001059, 6001055, 6001037, 6001032, 6001035, 6001038, 6001003, 6001004, 6001016, 6001018, 6001012 |
| CAAST1 | 5933877, 5933044, 5933888, 5933074, 5933045, 5933880, 5933042, 5933889, 5933887, 5933039, 5933884, 5933898, 5933054, 5939802, 5939805, 5933072, 5939801, 5933886, 5933060, 5933817, 5939807, 5939803, 5939037, 5939808, 5939811, 5939039, 5939032, 5933067, 5933024, 5933035, 5941017, 5933845, 5933876, 5933844, 5933812, 5933019, 5939806, 5933828, 5933854, 5939044, 5933846, 5939804, 5933806, 5933836, 5933837, 5939045, 5933857, 5937033, 5933802, 5933008, 5937017, 5937802, 5933028, 5933811, 5937024, 5937028, 5933875, 5937801, 5933810, 5937805, 5933834, 5933807, 5933861, 5933881, 5933895, 5933872, 5941802, 5933859, 5933032, 5933006, 5937803, 5941005, 5941813, 5937014, 5933805, 5933808, 5941016, 5939043, 5937021, 5933037, 5937041, 5931829, 5937010, 5931817, 5933801, 5933851, 5931820, 5931822, 5931815, 5935020, 5931816, 5935016, 5931842, 5933858, 5933848, 5931827, 5933839, 5931818, 5933840, 5931819, 5937005, 5931832, 5931823, 5933874, 5933809, 5931026, 5933868, 5931828, 5933878, 5933819, 5933831, 5933870, 5931814, 5933833, 5941803, 5935801, 5933813, 5931821, 5933841, 5933853, 5933873, 5933832, 5941015, 5933842, 5933852, 5933012, 5933015, 5933829, 5933855, 5931813, 5931824, 5933823, 5933850, 5933824, 5933825, 5933803, 5933866, 5933068, 5933865, 5933879, 5935803, 5935029, 5933849, 5941855, 5937022, 5933821, 5933882, 5933896, 5935010, 5935802, 5931034, 5933897, 5935018, 5935012, 5909850, 5909805, 5931831, 5933814, 5909819, 5939019, 5933070, 5941807, 5909815, 5931833, 5909847, 5937023, 5931826, 5939023, 5909806, 5941804, 5941806, 5909817, 5909836, 5907051, 5909014, 5907035, 5941868, 5907049, 5941873, 5941885, 5941012, 5941872, 5941811, 5941801, 5931844, 5909816, 5907055, 5907024, 5909809, 5907803, 5907041, 5941812, 5941014, 5909818, 5907807, 5941009, 5909876, 5903060, 5931812, 5907047, 5931843, 5941850, 5909852, 5909841, 5931032, 5941805, 5909808, 5903050, 5909883, 5909845, 5909875, 5909842, 5909848, 5907053, 5907809, 5909048, 5909016, 5941849, 5931012, 5909812, 5905054, 5907808, 5909009, 5907009, 5909804, 5907806, 5941810, 5909814, 5941010, 5909810, 5907028, 5907014, 5909807, 5907801, 5907802, 5931017, 5909843, 5939011, 5907026, 5953007, 5909832, 5905052, 5909027, 5931020, 5909032, 5941041, 5909034, 5907805, 5907022, 5909839, 5909060, 5909844, 5909834, 5907005, 5909837, 5941841, 5909833, 5903052, 5909838, 5909882, 5903032, 5939007, 5941821, 5909849, 5941882, 5903027, 5909825, 5909879, 5909036, 5909824, 5903058, 5909826, 5909821, 5909020, 5905042, 5909885, 5909884, 5909831, 5903019, 5909880, 5909835, 5953019, 5931021, 5903039, 5909881, 5909830, 5941883, 5905037, 5941019, 5909062, 5909827, 5909056, 5931801, 5931806, 5931006, 5931808, 5931802, 5931809, 5915075, 5915020, 5931807, 5905050, 5905032, 5941026, 5941025, 5909035, 5915840, 5909877, 5941831, 5915070, 5909878, 4815033, 5909052, 5953012, 5903056, 5915835, 5903043, 4815037, 5915034, 5915038, 5915816, 5903045, 5941808, 5915813, 5915039, 5915830, 5915036, 5903015, 5903023, 5915043, 5915804, 5915809, 5905030, 5941809, 5915046, 5915805, 5915001, 5905023, 5915806, 5941884, 5941013, 5941876, 5915002, 5915825, 5915065, 5915811, 5941829, 5915051, 5941828, 5915025, 5903041, 5915029, 5915807, 5905018, 5915004, 5915055, 5915808, 5905014, 5929022, 5929028, 5929018, 5915022, 5905026, 5929803, 5905005, 5915801, 5905009, 5915007, 5941879, 5901048, 5915062, 5941839, 5915803, 5915015, 5915011, 5929801, 5903011, 5929005, 5903047, 5929024, 5929026, 5927010, 5941021, 4815032, 5901040, 5941838, 5915810, 5915802, 5903010, 5929011, 5901039, 5901806, 4814028, 5941844, 5927016, 5941818, 5941827, 5901804, 5941817, 5941848, 4809810, 5941837, 5953046, 5927806, 5901046, 5927012, 5941027, 4814019, 5921014, 5941856, 5919817, 5901037, 5917805, 5919811, 5903017, 5901043, 5917029, 4815035, 5927008, 5927018, 5941881, 4818005, 5924054, 5927802, 5919809, 5927020, 5941852, 5921807, 5941835, 5921804, 5921010, 5921007, 5941866, 5919804, 5941039, 5917027, 5901028, 5921805, 5921008, 5919816, 5903004, 5919802, 5919021, 5919803, 5919017, 5921030, 5919801, 5903807, 5903013, 5924818, 5919008, 5921018, 5953048, 5953044, 4815023, 5917010, 5924820, 5917802, 5917005, 5919808, 5919013, 5924052, 5919015, 5919822, 5919818, 5919012, 4809002, 5921034, 5919049, 5919820, 5917803, 5921023, 5917801, 5953042, 5917015, 5917804, 5919815, 5919051, 5901803, 5921016, 5919043, 5921032, 5901022, 5919821, 5917021, 5921806, 5917030, 5919046, 5926005, 5917049, 5917034, 5926022, 5917047, 4815015, 5917812, 5917811, 5917040, 5919016, 5919812, 5926801, 4814003, 5941854, 5926010, 5924817, 5941851, 5921036, 5941864, 5926802, 5901802, 5917044, 5917041, 5953023, 5924804, 5926021, 5901035, 5919035, 5941871, 5901805, 5924840, 5926014, 5924812, 5941833, 5953801, 5924034, 4815802, 5917042, 5917054, 5923039, 5923033, 5923008, 4815013, 5923801, 4815030, 5923037, 5917052, 5917809, 5923802, 5923816, 5923822, 5926024, 5941880, 5919033, 4815027, 5917819, 5917056, 5941840, 4809809, 4809005, 5941845, 4814024, 4809806, 5953038, 5901003, 5941863, 5923035, 5941858, 4809015, 5919814, 4806036, 5941834, 4809010, 5917815, 5941862, 5923047, 5924042, 5951019, 5901012, 4806019, 4806031, 5923805, 5924039, 5901006, 5901019, 5941859, 5923809, 4806804, 5943037, 5941861, 5901017, 5943813, 4806803, 5951809, 4806009, 5951813, 5923814, 4806028, 5951007, 5945010, 4806011, 4811032, 4818015, 5923803, 4806008, 5901801, 5941860, 5923810, 4806032, 4815045, 4806014, 4806029, 4808024, 4806016, 5924835, 4806034, 4806026, 4815007, 4806001, 4806021, 5924025, 4811031, 5923049, 5923807, 5924803, 5923804, 4806012, 5923019, 4808006, 4808025, 5951017, 4808027, 4818002, 5943808, 4808013, 5943807, 5923823, 4806017, 4808012, 4808023, 5924048, 4808008, 4806006, 4808005, 5923813, 5943033, 4808044, 5923025, 5951802, 5923825, 5955021, 5923808, 5955003, 4808026, 5951806, 4808009, 4811026, 5943809, 4803026, 4808042, 5951009, 4808001, 4808028, 5951807, 4806022, 5923824, 4803016, 5945012, 4808011, 4813030, 5953050, 5951803, 5924030, 4806024, 4803011, 4819011, 5951840, 5951013, 5943008, 5951849, 4808029, 5943835, 4805044, 4811038, 5943837, 5945802, 4811024, 4819009, 4811039, 5951804, 4808038, 4805046, 4819012, 4803024, 4811005, 4808022, 5923806, 5945014, 4813002, 4803014, 4811004, 4808031, 4819006, 5924814, 4805018, 4811006, 4811020, 4811007, 5924029, 5953802, 4813010, 4803018, 5924836, 4813015, 4811022, 4811023, 4811001, 4803022, 4811003, 4811008, 4811041, 4811034, 4811009, 4819008, 4811802, 5943012, 4811045, 4811806, 4818816, 5924833, 4819815, 4811044, 4811042, 4811807, 5945803, 5951805, 4805041, 4805048, 4811021, 4813001, 5951826, 5924805, 5951824, 4803021, 4813006, 4803801, 5945006, 4808039, 4819014, 4818018, 4813811, 4805049, 4805006, 4813029, 4808032, 4805022, 4818818, 4805042, 5951031, 4813005, 4803003, 4805011, 5951818, 4813012, 4808002, 4813008, 4813011, 4813007, 4811046, 5943817, 4813009, 4808004, 5943804, 4811803, 5943815, 5943031, 4803019, 4811012, 5951819, 4808813, 5943023, 4811019, 4808812, 4805004, 5951823, 4805021, 4803008, 5951810, 4805012, 4811801, 5943806, 4813014, 4811048, 4808034, 4803006, 4808811, 5951822, 4805002, 5943017, 4805802, 4803803, 5924806, 4805001, 5951022, 4811018, 5943816, 5951801, 4811049, 4813013, 5924813, 4811011, 4802016, 5953033, 5951827, 5951815, 5951844, 4811002, 4805009, 4813003, 4811804, 4813017, 4811016, 4813018, 4802014, 5955023, 4813016, 4805038, 4805036, 5955005, 4813019, 4817024, 4803802, 4805019, 4811805, 5951028, 5955014, 4810002, 4807021, 5951847, 4805026, 4807028, 5951820, 5955010, 4803004, 4811013, 5943836, 4802013, 4810004, 4811062, 4807027, 5951833, 5951825, 4807026, 4811061, 4805031, 5943027, 4805008, 4819056, 4810012, 4819058, 4819054, 4802018, 5951814, 4819059, 4802012 |
| CAAST2 | 3560049, 3560055, 3558085, 3560046, 3560054, 3558080, 3558097, 3560053, 3560085, 3560093, 3560098, 3560059, 3560081, 3560090, 3560086, 3560102, 3560056, 3558065, 3560034, 3560080, 3558090, 3560100, 3560032, 3560097, 3560084, 3560104, 3560042, 3560079, 3560001, 3560096, 3560075, 3560027, 3560077, 3560088, 3560057, 3558076, 3560095, 3558069, 3560071, 3558095, 3560024, 3558077, 3560021, 3558075, 3560067, 3558100, 3560070, 3560076, 3560052, 3560058, 3558064, 3558044, 3558041, 3558068, 3559001, 3558034, 3558067, 3558028, 3558019, 3559090, 3558063, 3559066, 3558004, 3558011, 3560004, 3558016, 3560010, 3560089, 3560005, 3560083, 3560066, 3558003, 3558012, 3559064, 3560061, 3559065, 4619079, 4619051, 3560008, 3558051, 3560063, 3560069, 3559060, 3558001, 4622056, 3560007, 3558054, 3559048, 3559068, 3559061, 3559069, 3559063, 3559062, 3559012, 3559053, 3559052, 3559051, 4622048 |
| CAAST3 | 3526011, 3526032, 3526003, 3526014, 3526043, 3526028, 3526037, 3526053, 3526057, 3526047, 3526021, 3526065, 3528018, 3528037, 3528035, 3525005, 3524001, 3529021, 3524002, 3529020, 3521005, 3524009, 3529006, 3520005, 3529005 |
| CAAST4 | 3551094, 3551100, 3551044, 3551034, 3551028, 3551027, 3557073, 3551026, 3557040, 3557072, 3557026, 3557035, 3557038, 3551021, 3557039, 3557028, 3551045, 3551006, 3557041, 3557071, 3557094, 3551041, 3551001, 3557021, 3551017, 3557004, 3557019, 3551042, 3557006, 3557001, 3552023, 3551011, 3557016 |
| CAAST5 | 3530010, 3530004, 3530013 |
| ABAST1 | 4806804, 4806016 |
| BCAST1 | 5915022, 5915803, 5915807, 5915808 |
| BCAST2 | 5933805, 5933006, 5933801, 5933811, 5933807, 5933809, 5933012, 5933808, 5933806, 5933008, 5933802, 5933859, 5933861, 5933035, 5933851, 5909847, 5909819, 5909815, 5909850, 5933834, 5909805, 5933803, 5909806, 5933881, 5933895, 5909817, 5933897, 5909836, 5933896, 5933857, 5933882, 5933810, 5933849, 5933879, 5933865, 5933823, 5933825, 5933824, 5933015, 5933829, 5933866, 5933832, 5933850, 5933831, 5933855, 5933853, 5933852, 5933833, 5933842, 5933813, 5933873, 5909816, 5933037, 5933841, 5909809, 5933846, 5933868, 5909014, 5933870, 5933854, 5907055, 5933878, 5933828, 5907024, 5933874, 5933876, 5933837, 5933042, 5935020, 5909818, 5933840, 5933848, 5933839, 5933019, 5933880, 5909876, 5933039, 5933844, 5933060, 5935018, 5933845, 5933817, 5907051, 5933858, 5909841, 5935029, 5909808, 5935802, 5933024, 5931827, 5909016, 5935803, 5907035, 5909812, 5937017, 5909009, 5933812, 5931832, 5931815, 5907807, 5935016, 5935010, 5935801, 5909804, 5933875, 5933044, 5931820, 5909814, 5937014, 5933836, 5937803, 5937801, 5907049, 5931823, 5931822, 5931828, 5909810, 5907803, 5933872, 5909807, 5933898, 5931026, 5931814, 5933877, 5931816, 5907053, 5931842, 5939039, 5931819, 5933045, 5909842, 5931821, 5909048, 5939804, 5907041, 5931817, 5931824, 5907809, 5933884, 5909843, 5909848, 5931818, 5939806, 5933054, 5937805, 5935012, 5937010, 5909832, 5907808, 5909027, 5909034, 5909032 |
| BCAST3 | 5909878, 5909035, 5909052, 5915840, 5909877, 5909056, 5915825, 5915835, 5915001, 5909062, 5915816, 5909881, 5915075, 5909880, 5915002, 5915813, 5915830, 5909879, 5915809, 5909835, 5909826, 5909885, 5909821, 5909824, 5909827, 5915070, 5909882, 5909825, 5909830, 5909884, 5909831, 5909020, 5915004 |
| ONAST1 | 3526003, 3526011, 3526043, 3526032, 3526037, 3526028, 3526014, 3526047, 3526053, 3526057, 3526021, 3526065, 3528018, 3528037, 3524001, 3524002, 3525005, 3528035, 3529021, 3529020, 3521005, 3520005, 3524009, 3529006 |
| ONAST2 | 3560091, 3560078, 3560096, 3560079, 3560100, 3560088, 3560051, 3560086, 3560075, 3560090, 3560085, 3560081, 3560093, 3560098, 3560052, 3560095, 3560050, 3556093, 3560053, 3560059, 3560097, 3560076, 3560049, 3556106, 3560055, 3556094, 3560104, 3556096, 3558076, 3558085, 3556092, 3560071, 3560054, 3560102, 3560080, 3558097, 3556095, 3558068, 3558067, 3560046, 3558075, 3558077, 3556076, 3556077, 3558090, 3558080, 3560070, 3558065, 3556073, 3557096, 3556070, 3558069, 3558066, 3556066, 3556056, 3560077, 3560067, 3556052, 3558064, 3556048, 3558063, 3558044, 3558059, 3558060, 3558051, 3558054, 3560056, 3558041, 3558061, 3560042, 3560034, 3558062, 3560032, 3557091, 3556042, 3558034, 3560084, 3556102, 3557079, 3558028, 3560001, 3556100, 3558100, 3558095, 3560027, 3560057, 3556031, 3558004, 3556027, 3560024, 3557078, 3557076, 3557095, 3558019, 3560021, 3558011, 3558003, 3560058, 3558016, 3556033, 3556014, 3558012, 3552058, 3556091, 3552092, 3558001, 3552054, 3552053, 3559001, 3556098, 3560061, 3560089, 3560010, 3552052, 3554057, 3554068, 3559090, 3560066, 3559066, 3560004, 3554091, 3554056, 3560005, 3560083, 3554066, 3560069, 3552093, 3554058, 3560008, 3559064, 3559065, 3559060, 3554062, 3560063, 3554094, 3560007, 3559048, 3554054, 3559068, 3554042, 3560068, 3559061, 3554052, 3554044, 3559069, 3560064, 3554049, 3559063, 3559053, 3560082, 3559062, 3559052, 3559012, 3559051, 3559047, 3560065, 3554036, 3554034, 3560060, 3559011, 3559024, 3554038, 3559016, 3554032, 3554024, 3559019, 3559031, 3554026, 3559026, 3554029, 3554021, 3557077, 3554014, 3554020, 3559040, 3554008, 3554001, 3559042, 3557066, 3557074, 3557075, 3557061, 3554006, 3557051, 3557011, 3557016, 3548072, 3557014, 3548069, 3557019, 3553040, 3557021, 3557041, 3557038, 3557008, 3557006, 3557035, 3557028, 3557026, 3557004, 3553005, 3557001, 3557073, 3557040, 3552031, 3552028, 3557072, 3557039, 3552023, 3552013, 3552026, 3552051, 3557071, 3557094, 3548094, 3551034, 3551100, 3551044, 3552004, 3551040, 3548055, 3551094, 3551026, 3551027, 3551041, 3551021, 3548073, 3552036, 3551017, 3551028, 3552001, 3551042, 3551045, 3548044, 3551006, 3551011, 3549076, 3549077, 3548034, 3548019, 3551043, 3549066, 3548027, 3551001, 3549075, 3548022, 3548021, 3549071, 3549096, 3548031, 3549060, 3549078, 3548013, 3549095, 3549054, 3549056, 3549039, 3547098, 3549048, 3549051, 3549046, 3549072, 3549043, 3549022, 3549019, 3549024, 3549036, 3549028, 3549031, 3549005, 3548091, 3549018, 3541069, 3549032, 3549014, 3547096, 3549012, 3549073, 3547090, 3549003, 3547076, 3544042, 3541060, 3544073, 3544027, 3544053, 3546018, 3547064, 3548001, 3547075, 3541055, 3544065, 3544071, 3547033, 3547030, 3543069, 3544018, 3542053, 3543070, 3547070, 3547056, 3546024, 3547037, 3541057, 3543072, 3542059, 3542047, 3544002, 3543068, 3543074, 3512076, 3547035, 3543071, 3541045, 3543015, 3546015, 3541043, 3547020, 3547043, 3542037, 3542045, 3547046, 3543031, 3547048, 3512065, 3543050, 3546005, 3541024, 3543064, 3512061, 3512058, 3543009, 3543023, 3543019, 3542015, 3543052, 3547008, 3547003, 3541032, 3543005, 3547002, 3542029, 3542004, 3541015, 3512054, 3512051, 3543042, 3515044 |
| ONAST3 | 3523025, 3523009, 3522001, 3530035, 3523017, 3523033, 3523008, 3522010, 3523050, 3530016, 3530013, 3522014, 3522008, 3523001, 3530027, 3530010 |
| NSAST1 | 1216011, 1216014, 1217020, 1216006, 1216001, 1217030, 1218003, 1215008, 1215001, 1218001, 1217008, 1215002, 1213008, 1215006 |
| QCAST1 | 2480050, 2480055, 2480060, 2480065, 2480070, 2480045, 2482005, 2480037, 2480027, 2480078, 2480085, 2480010, 2482010, 2480020, 2480005, 2480090, 2480015, 2481017, 2482015 |

Notes: The shape file used for the map, CSDUIDs, and CSD names can be found here: https://www12.statcan.gc.ca/census-recensement/alternative_alternatif.cfm?l=eng&dispext=zip&teng=lcsd000b16a_e.zip&k=%20%20%20%2047761&loc=http://www12.statcan.gc.ca/census-recensement/2011/geo/bound-limit/files-fichiers/2016/lcsd000b16a_e.zip
